# Interactions between species introduce spurious associations in microbiome studies

**DOI:** 10.1371/journal.pcbi.1005939

**Published:** 2018-01-16

**Authors:** Rajita Menon, Vivek Ramanan, Kirill S. Korolev

**Affiliations:** 1 Department of Physics, Boston University, Boston, Massachusetts, United States of America; 2 BRITE Bioinformatics REU Program, Boston University, Boston, Massachusetts, United States of America; 3 Department of Biology and Computer Science, Swarthmore College, Swarthmore, Pennsylvania, United States of America; 4 Graduate Program in Bioinformatics, Boston University, Boston, Massachusetts, United States of America; University of Chicago, UNITED STATES

## Abstract

Microbiota contribute to many dimensions of host phenotype, including disease. To link specific microbes to specific phenotypes, microbiome-wide association studies compare microbial abundances between two groups of samples. Abundance differences, however, reflect not only direct associations with the phenotype, but also indirect effects due to microbial interactions. We found that microbial interactions could easily generate a large number of spurious associations that provide no mechanistic insight. Using techniques from statistical physics, we developed a method to remove indirect associations and applied it to the largest dataset on pediatric inflammatory bowel disease. Our method corrected the inflation of p-values in standard association tests and showed that only a small subset of associations is directly linked to the disease. Direct associations had a much higher accuracy in separating cases from controls and pointed to immunomodulation, butyrate production, and the brain-gut axis as important factors in the inflammatory bowel disease.

## Author summary

Microbiomes are important for better health, sustainable agriculture, and climate management. Since experimental studies of natural microbial communities are often challenging, microbiome wide association studies (MWAS) have been the primary method to reveal the connection between specific microbes and host phenotype. MWAS have established that many diseases are associated with a complex dysbiosis driven by a large number of microbes. We show that many of these associations may not reflect a mechanistic link with the disease, but arise instead due to interspecific interactions such as cross-feeding and competition for resources. We also propose a method grounded in the maximum entropy models of statistical physics to separate direct from indirect associations. Using both synthetic and real microbiome data, we show that this method detects only direct associations, identifies most predictive features of microbiomes, and avoids p-value inflation. We demonstrate the power of this method on one of the largest microbiome data sets on inflammatory bowel disease.

## Introduction

Microbes are essential to any ecosystem be it the ocean or the human gut. The sheer impact of microbial processes has however been underappreciated until the advent of culture-independent methods to assess entire communities *in situ*. Metagenomics and 16S rRNA sequencing identified significant differences in microbiota among hosts, and experimental manipulations established that microbes could dramatically alter host phenotype [1–8]. Indeed, anxiety, obesity, colitis, and other phenotypes can be transmitted between hosts simply by transplanting their intestinal flora [9–13].

New tools and greater awareness of microbiota triggered a wave of association studies between microbiomes and host phenotypes. Microbiome wide association studies (MWAS) have been carried out for diabetes, arthritis, cancer, autism and many other disorders [14–23]. MWAS clearly established that each disease is associated with a distinct state of intestinal dysbiosis, but they often produced conflicting results and identified a very large number of associations both within and across studies [14, 19, 21, 23–26]. For example, a recent study on inflammatory bowel disease (IBD) reported close to 100 taxa associated with IBD [25], a number that is fairly typical [14]. Such long lists of associations defy simple interpretation and complicate mechanistic follow-up studies because one needs to examine the role of almost every species in the microbiota. In fact, one can argue that MWAS are most useful when they can identify a small network of taxa driving the disease.

Although extensive dysbiosis might reflect the multifactorial nature of the disease, it is also possible that MWAS detect spurious associations because their statistical methods fail to account for some important aspects of microbiome dynamics. One such aspect is the pervasive nature of microbial interactions: species compete for similar resources, rely on cross-feeding for survival, and even produce their own antibiotics [27–37]. Hence, microbial abundances must be correlated with each other, and even a simple change in host phenotype could manifest as collective responses by the microbiota. Traditional MWAS, however, completely neglect this possibility because they treat each species as an independent manifestation of host phenotype. As a result, MWAS cannot distinguish taxa directly linked to disease from taxa that are affected only through their interactions with other species.

The main conclusion of this paper is that realistic microbial interactions produce a large number of spurious associations between particular members of the microbiome and phenotypes. Many of these indirect associations can be removed by a simple procedure based on maximum entropy models from statistical physics [38, 39]. We dubbed this approach Direct Association Analysis, or DAA for short.

When applied to the largest MWAS on IBD, DAA shows that many of the previously reported associations could be explained by interspecific interactions rather than the disease. At the genus and species level, the direct associations include only *Roseburia*, *Faecalibacterium prausnitzii*, *Bifidobacterium adolescentis*, *Blautia producta*, *Turicibacter*, *Oscillospira*, *Eubacterium dolichum*, *Aggregatibacter segnis*, and *Sutterella*. Some of these associations are well-known [40–47], while others have received little attention in IBD research. The phenotypes of the taxa directly linked to disease suggest that immunomodulation, butyrate production, and the brain-gut interactions play an important role in the etiology of IBD.

Compared to traditional MWAS, DAA corrected the inflation of p-values responsible for the large number of spurious associations and identified taxa most informative of the diagnosis. We found that directly associated taxa are much better at discriminating between cases and controls than an equally-sized subset of indirect associations. In fact, direct associations have the same potential to discriminate between health and disease as the entire set of almost a hundred associations detected by conventional methods.

## Results

**Fig 1:**
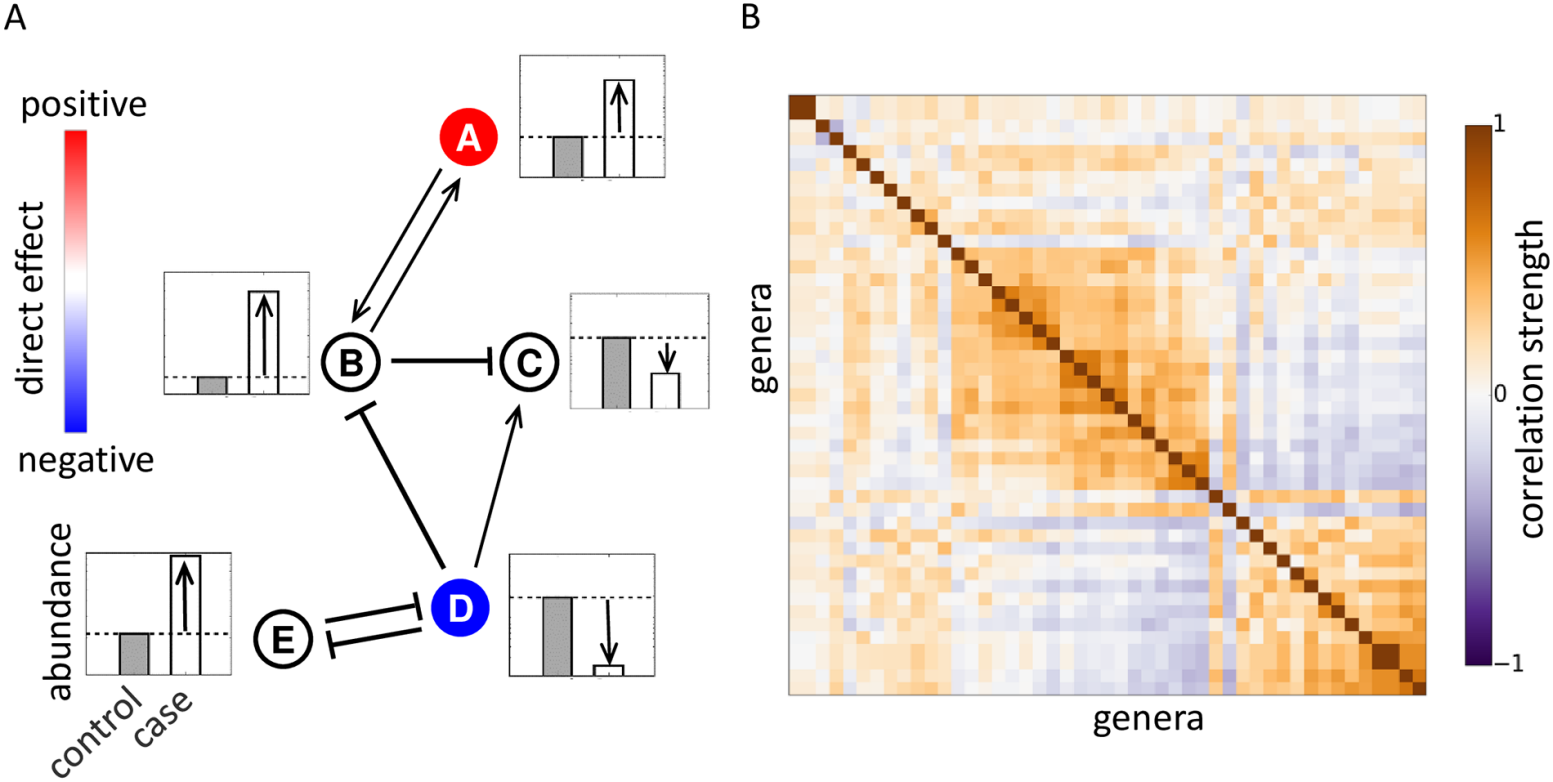
Microbial interactions generate spurious associations. (**A**) A hypothetical interaction network of five species together with their dynamics in disease. Only two species (shown in color) are directly linked to host phenotype. These directly-linked species inhibit or promote the growth of the other members of the community (shown with arrows). As a result, all five species have different abundances between case and control groups. (**B**) Microbial interactions are visualized via a hierarchically-clustered correlation matrix computed from the data in Ref. [21]. We used Pearson’s correlation coefficient between log-transformed abundances to quantify the strength of co-occurrence for each genus pair. Dark regions reflect strong interspecific interactions that could potentially generate spurious associations. See S1 Text for the list of 47 most prevalent genera included in the plot.

Traditional MWAS detect species with significantly different abundances between case and control groups. Some changes in the abundances are directly associated with the disease while others are due to microbial interactions. The emergence of indirect changes in abundance is illustrated in Fig 1A for a hypothetical network of five species. Only two species A and D are directly linked to the disease. However, strong interactions make the abundances of all five species differ between control and disease groups. For example, the mutualistic interaction between A and B helps B grow to a higher density following the increase in the abundance of A. The expansion of B in turn inhibits the growth of C and reduces its abundance in disease. Strong mutualistic, competitive, commensal, and parasitic interactions have been demonstrated in microbiota [27–37], and Fig 1B shows that almost every species present in the human gut participates in a strong interaction. Thus, the propagation of abundance changes from directly-linked to other species could pose a significant challenge for MWAS. To test this hypothesis, we turned to a minimal mathematical model of microbiota composition.

### Maximum entropy model of microbiota composition

A quantitative description of interspecific interactions and their effect on MWAS requires a statistical model of host-associated microbial communities. Ideally, such a model would describe the probability to observe any microbial composition, but the amount of data even in large studies is only sufficient to determine the means and covariances of microbial abundances. This situation is common in the analysis of biological data and has been successfully managed with the use of maximum entropy distributions [38]. These distributions are chosen to be as random as possible under the constraints imposed by the first and second moments. Maximum entropy models introduce the least amount of bias and reflect the tendency of natural systems to maximize their entropy [48, 49]. In other contexts, these models have successfully described the dynamics of neurons, forests, flocks, and even predicted protein structure and function [50–54]. In the context of microbiomes, a recent work derived a maximum entropy distribution for microbial abundances using the principle of maximum diversity [55].

We show in S1 Text that the maximum entropy distribution of microbial abundances *P*({*l*_*i*_}) takes the following form

$$P\left(\left\{l_{i}\right\}\right)=\frac{1}{Z}e^{\sum_{i}h_{i}l_{i}+\frac{1}{2}\sum_{ij}J_{ij}l_{i}l_{j}}$$ (1)

where *l*_*i*_ is the log-transformed abundance of species *i*, *h*_*i*_ represents the direct effect of the host phenotype on species *i*, and *J*_*ij*_ describes the interaction between species *i* and *j*; the factor of 1/*Z* is the normalization constant. The log-transformation of relative abundances alleviates two common difficulties with the analysis of the microbiome data. The first difficulty is the large subject-to-subject variation, which is much better captured by a log-normal rather than a Gaussian distribution; see S1 Fig, and Ref. [25]. The second difficulty arises from the fact that the relative abundances must add up to one. This constraint is commonly known as the compositional bias because it leads to artifacts in the statistical analysis [56–58]. The log-transformation is an essential first step in most methods that account for the compositional bias including the widely advocated log-ratio transformation [56–59], which includes additional steps that are not relevant in the context of Eq (1). In S1 Text, we generalize Eq (1) to account for the constraint imposed by data normalization and show that our conclusions are not affected by the compositional bias.

The key prediction of Eq (1), see S1 Text, is that *h* and mean microbial abundances *m*_*i*_ = 〈*l*_*i*_〉 are related by *m* = *J*^−1^*h*. Because of interspecific interactions, *J* is not diagonal, and, therefore, a change in one component of *h* affects the abundances of many species. We show below that this nontrivial cause-effect relationship gives rise to spurious associations in both synthetic and real microbiome data.

### Testing for spurious associations in synthetic data

**Fig 2:**
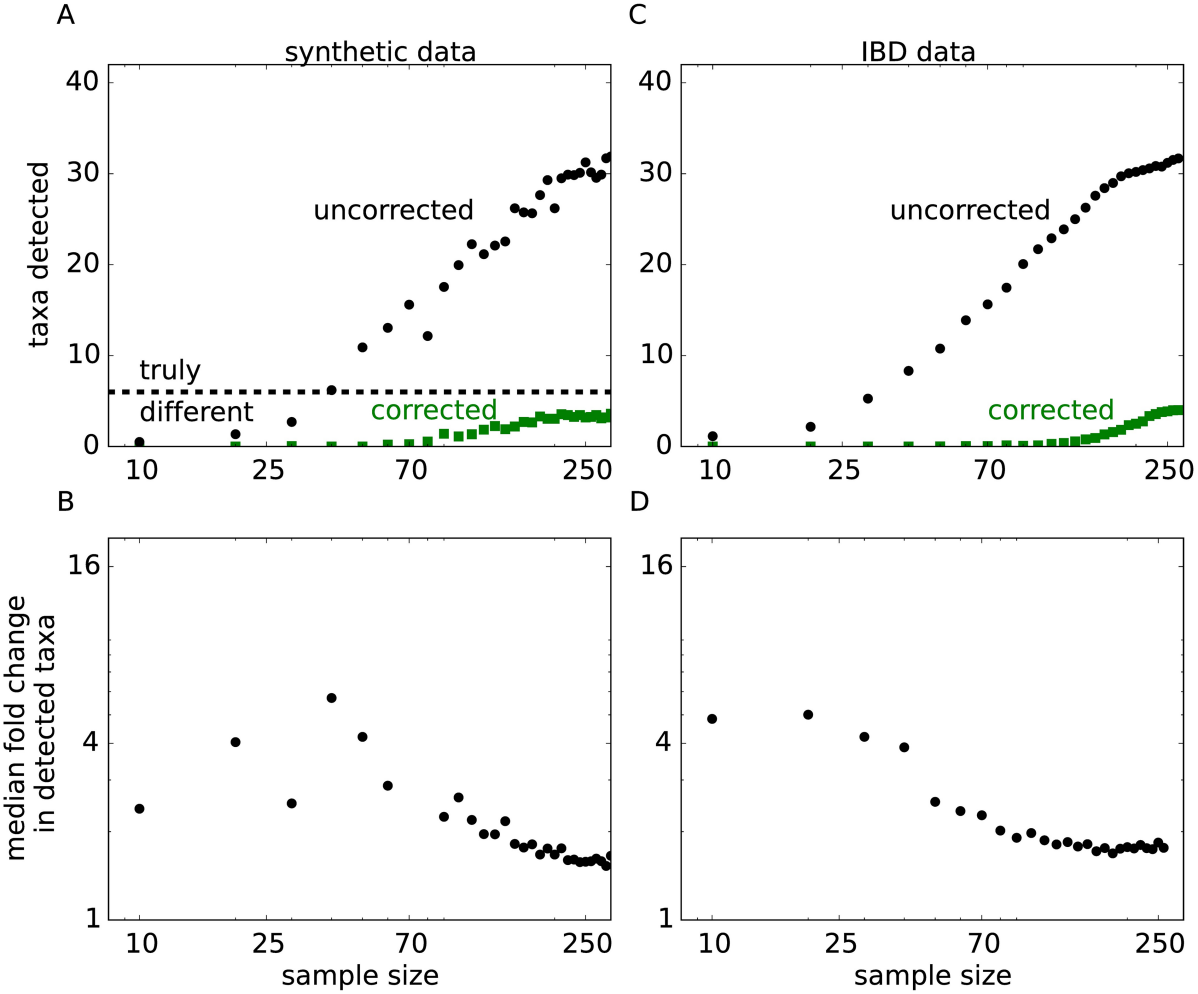
Signatures of indirect associations in synthetic and IBD data sets. The synthetic data set was generated to match the statistical properties of the IBD data set from Ref. [21], but with a predefined number of 6 directly associated taxa (See S1 Text). (**A**) In synthetic data, DAA identifies no spurious association and detects 4 out of 6 directly associated genera. All 6 genera and no false positives are detected when the sample size is increased further (S9 Fig). In sharp contrast, a large number of spurious associations is observed for metrics that rely on changes in abundance between cases and controls and do not correct for microbial interactions. The number of false positives grows rapidly with statistical power until all taxa are reported as significantly associated with the disease. (**B**) All spurious associations show substantial differences between cases and controls and, therefore, cannot be discarded based on their effect sizes. To quantify the effect size, we estimated the magnitude of the fold change for each genus. Specifically, we first computed the difference in the mean log-abundance between cases and controls and then exponentiated the absolute value of this difference. The plot shows how the median effect size for significantly associated genera depends on the sample size. Larger samples sizes result in much higher number of associations, but only a small drop in the typical effect size. (**C**) and (**D**) are the same as (A) and (B), but for the IBD data set. The results are consistent between the two data sets suggesting that most associations detected by traditional MWAS are spurious. The complete list of indirect associations inferred from the IBD data set is shown in S1 Text, and the results for different synthetic data sets are shown in S14 Fig.

We obtained realistic model parameters from one of the largest case-control studies previously reported in Ref. [21]. The samples were obtained from mucosal biopsies of 275 newly diagnosed, treatment-naive children with Crohn’s disease (a subtype of IBD) and 189 matched controls. Microbiota composition was determined by 16S rRNA sequencing with about 30,000 reads per sample. From this data, we inferred the interaction matrix *J* and the typical changes in microbial abundances associated with the disease for 47 most prevalent genera (Methods and S1 Text). Even though the number of data points significantly exceeds the number of free parameters in the model, overfitting could still be a potential concern. However, overfitting is unlikely to affect our main conclusions because they depend only on the overall statistical properties of *J* rather than on the precise knowledge of every interaction. In fact, none of our results changed when we analyzed only about half of the data set (Fig 2 and S12 Fig). To improve the quality and robustness of the inference procedure, we also used the spectral decomposition of *J* to remove any interaction patterns that were not strongly supported by the data; see Methods and S1 Text for further details.

To determine the effect of microbial interactions on conventional MWAS analysis, we generated synthetic data with a known number of direct associations. The data for the control group was used without modification from Ref. [21]. The disease group was generated using Eq (1) with the same values of *h* and *J* as in the control group, except we modified the values of *h* for 6 representative genera (see S1 Text). We also generated two other synthetic data sets with smaller and larger effect sizes. The results for all three data sets were very similar (S1 Text).

The synthetic data was further subsampled to several sample sizes in order to simulate variation in statistical power between different studies. For an ideal method, the number of detected associations should increase with the cohort size, but eventually saturate once all 6 directly associated genera are discovered. In contrast to this expectation, the number of associations detected by the conventional approach increased rapidly with the sample size until almost all genera were found to be statistically associated with the disease in our synthetic data. At this point, traditional MWAS completely lost the power to identify the link between the phenotype and microbiota. Unbounded growth in the number of detections was also observed for the real data (Fig 2C) suggesting that many previously reported associations between microbiota and IBD could be indirect.

Are spurious associations simply an artifact of our ability to detect even minute differences between cases and controls? Fig 2B and 2D show that this was not the case. The median effect size declined only moderately with the number of associations, and most associations corresponded to about a factor of two difference in the taxon abundance. Thus, spurious associations are not weak and could not be discarded based on their effect size.

### Direct association analysis (DAA)

**Fig 3:**
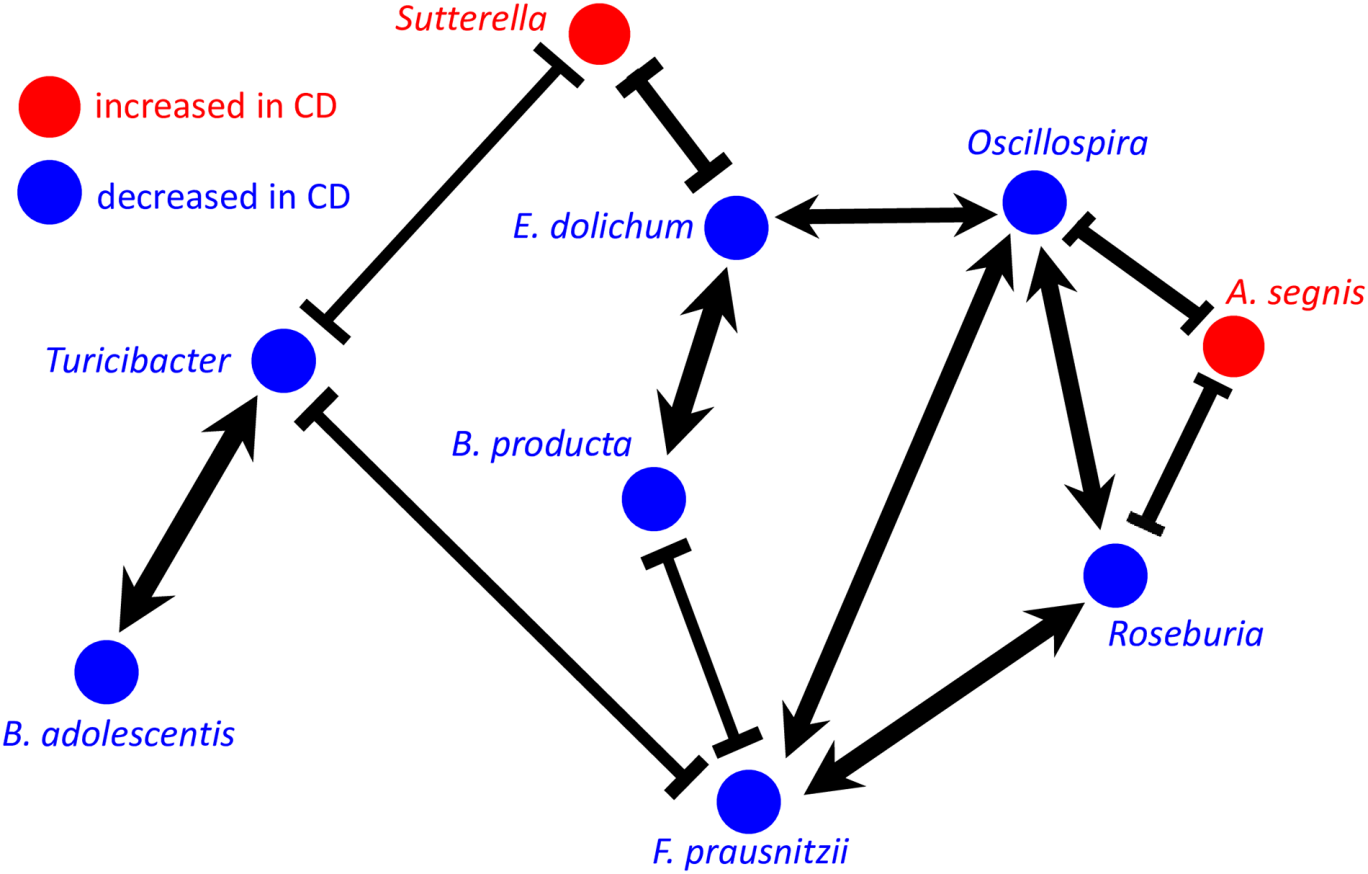
Network of direct associations with Crohn’s Disease. Five species and four genera were found to be significantly associated with Crohn’s Disease (*q* < 0.05) after correcting for microbial interactions (S1 and S4 Figs). The links correspond to significant interactions (*q* < 0.05) between the taxa with *J*_*ij*_ > 0.27 or *J*_*ij*_ < −0.15; the width of the arrows reflects the strength of the interactions. For comparison, the correlation-based network for directly associated taxa is shown in S7 and S5 Figs, and a complete summary of correlations and interactions for all species pairs is provided in S1 Text.

Fortunately, the maximum entropy model provides a straightforward way to separate direct from indirect associations. Since direct effects are encoded in *h*, MWAS should be performed on *h* rather than on *l*. This simple change in the statistical analysis correctly recovered 4 out of 6 directly associated taxa in the synthetic data and yielded no indirect associations even for large cohorts (Fig 2A and S9 Fig). Similarly good performance was found for the two other synthetic data sets (S14 Fig). For the IBD data, DAA also identified a much smaller number of associations compared to traditional MWAS analysis and showed clear saturation at large sample sizes (Fig 2B). Direct associations with IBD are summarized in Fig 3 at the genus and species levels, and the entire phylogenetic tree of direct associations is shown in S4 Fig and in S1 Text.

In addition to associations, DAA also infers the network of direct microbial interactions (Fig 3, S5 and S6 Figs). While the sample size is insufficient to accurately infer the interactions between every pair of microbes, strong interactions and the overall properties of the interaction network can nevertheless be determined from the data. The interactions inferred by DAA describe only direct effects of the species on each other and do not include induced correlations present in the correlation matrix. That is, DAA controls for the fact that species A and C could be correlated because both interact with species B, but not with each other (Fig 1A). The ability of maximum entropy models to separate direct from indirect interactions has been the primary reason for their applications to biological data [50–54]. Similar to these previous studies, many direct interactions reported in Fig 3 are also present in the correlation-based network, but DAA removes some induced interactions and identifies a few interactions that are not evident in the correlation data; see S5 and S6 Figs. Overall, the interaction network is much sparser than the correlation network in Fig 1B. In S1 Text, we also compare the results from DAA and SparCC [56], a widely used package to infer correlation networks from microbiome data (S6 Fig).

**Fig 4:**
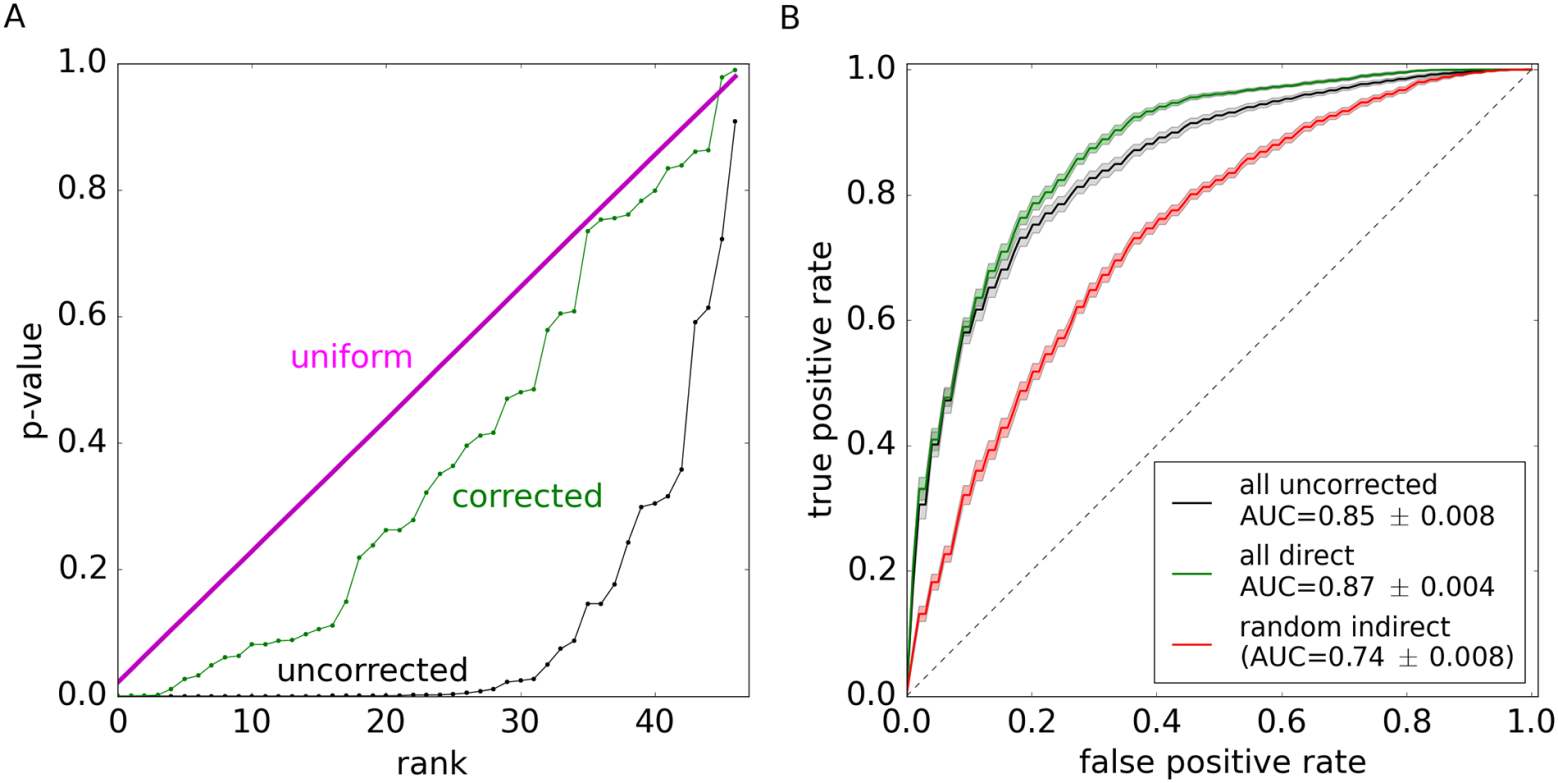
Direct associations analysis corrects p-value inflation and retains diagnostic accuracy. (**A**) The distribution of p-values in DAA closely follows the expected uniform distribution. Because conventional MWAS does not correct for microbial interactions, it yields an excess of low p-values, which is a strong signature of indirect associations. For both methods, p-values were computed using a permutation test. The expected uniform distribution was obtained by sampling from a generator of uniform random numbers. The ranked plot of p-values visualizes their cumulative distribution functions; this is a variant of a Q-Q plot. (**B**) Direct associations are a small subset of all associations with IBD (see S4 Fig), yet they retain full power in classifying samples as cases or controls. In contrast, the classification power is substantially reduced for an equally-sized subset of randomly-chosen indirect associations. In each case, we used sparse logistic regression to train a classifier on 80% of the data and tested its performance on the remaining 20% (Methods). The shaded regions show one standard deviation obtained by repeated partitioning the data into training and validation sets. Identical results were obtained with a random forest [64, 65] and support vector machine [66] classifiers (S8 Fig)

To demonstrate that DAA isolates direct effects from collective changes in the microbiota, we examined the p-value distribution in this method. The distribution of p-values is commonly used as a diagnostic tool to test whether a statistical method is appropriate for the data. In the absence of any associations, p-values must follow a uniform distribution because the null hypothesis is true [60]. A few strong deviations from the uniform distribution signal true associations [61]. In contrast, large departures from the uniform distribution typically indicate that the statistical method does not account for some properties of the data, for example, population stratification in the context of genome wide association studies [62, 63]. Fig 4A compares the distribution of p-values for DAA and a conventional method in MWAS. Consistent with our hypothesis that interspecific interactions cannot be neglected, conventional analysis generates an excess of low p-values and, as a result, a large number of potentially indirect associations. In contrast, the distribution of p-values from DAA matches the expected uniform distribution and, thus, provides strong support for our method.

## Discussion

The primary goal of MWAS is to guide the study of disease etiology by detecting microbes that have a direct effect on the host. These direct effects could be very diverse and include secretion of toxins, production of nutrients, stimulation of the immune system, and changes in mucus and bile [67, 68]. In addition to the host-microbe interactions, the composition of microbiota is also influenced by the interspecific interactions among the microbes such as competition for resources, cross-feeding, and production of antibiotics [27–37]. In the context of MWAS, microbial interactions contribute to indirect changes in microbial abundances, which are less informative of the disease mechanism and are less likely to be valuable for follow-up studies or in interventions. Here, we estimated the relative contribution of indirect associations to MWAS and showed how to isolate direct from indirect associations.

Our main result is that interspecific interactions are sufficiently strong to generate detectable changes in the abundance of many microbes that are not directly linked to host phenotype. As a result, conventional approaches to MWAS detect a large number of spurious associations and produce inflated p-values that do not match their expected distribution (Fig 4A). These challenges are resolved by Direct Association Analysis (DAA), which uses maximum entropy models to explicitly account for interspecific interactions. We applied DAA to a large data set of pediatric Crohn’s disease and found that it restores the distribution of p-values and substantially simplifies the pattern of dysbiosis while retaining full classification power of a conventional MWAS.

The relatively simple dysbiosis identified by DAA in IBD has strong support in the literature and offers interesting insights into disease etiology. Four of the taxa identified by our method have a well-established role in IBD: *B. adolescentis*, *F. prausnitzii*, *B. producta*, and *Roseburia*. They have been repeatedly found to have lower abundance in both Crohn’s disease and ulcerative colitis [40–47], and several studies have demonstrated their ability to suppress inflammation and alleviate colitis [43, 69–73]. *Bifidobacterium* species occupy a low trophic level in the gut and ferment complex polysaccharides such as fiber [74, 75]. Fermentation products include lactic acid, which promotes barrier function, and maintains a healthy, slightly acidic environment in the colon [76]. Due to these properties *Bifidobacterium* species are commonly used as probiotics [74]. *F. prausnitzii*, *Blautia producta* and *Roseburia* occupy a higher trophic level and ferment the byproducts of polysaccharides digestion into short-chain fatty acids (SCFA), which are an important energy source for the host [42, 43, 77, 78].

The ability of DAA to detect taxa strongly associated with IBD is reassuring, but not surprising. What is surprising is that many strong associations are classified as indirect by our method. For example, *Roseburia* and *Blautia* are the only genera of *Lachnospiraceae* that DAA finds to be directly linked to the disease. In sharp contrast, traditional MWAS report seven genera in this family that are strongly associated with IBD [25]. All seven genera are involved in SCFA metabolism, but their specializations differ. Species in *Blautia* genus are major producers of acetate, a SCFA that is commonly involved in microbial crossfeeding [79, 80]. In particular, many species extract energy from acetate by converting it into butyrate, another SCFA that plays a major role in gut health by nourishing colonocytes and regulating the immune function [77, 80]. *Roseburia* genus specializes almost exclusively in the production of butyrate and acts as a major source of butyrate for the host [77, 81]. Thus, our findings suggest that butyrate production plays an important role in IBD etiology and that the dysregulation of this process is directly linked to the depletion of *Roseburia* and possibly *Blautia*.

The important role of butyrate is further supported by our detection of *E. dolichum* and *Oscillospira*, which are known to produce butyrate [82–84]. The latter taxon has not been detected in three independent analyses of this IBD data set [21, 25, 85] presumably because its involvement is masked by indirect associations and interactions with other microbes. Several other studies support this DAA finding and confirm that *Oscillospira* is suppressed in IBD [86, 87]. *Oscillospira* was also found to be positively associated with leanness and negatively associated with the inflammatory liver disease [88–90]. The interactions between *Oscillospira* and the host appears to be quite complex and involve the consumption of host-derived glycoproteins including mucin, production of SCFA, and modulation of bile-acid metabolism [84, 91]. The latter interaction was suggested to be a major factor in the protective role of *Oscillospira* against infections with *Clostridium difficile* [91–93].

The final taxon that was suppressed in IBD is *Turicibacter*. This genus is not very well characterized, and few MWAS studies point to its involvement in IBD [21, 25, 94]. Two studies in animal models, however, directly looked into the connection between IBD and *Turicibacter* [95, 96]. The first study found that iron limitation eliminates colitis in mice while at the same time restoring the abundance of *Turicibacter*, *Bifidobacterium*, and four other genera [95]. The second study identified *Turicibacter* as the only genus that is fully correlated with immunological differences between mice resistant and susceptible to colitis: high abundance of *Turicibacter* in the colon predicted high levels of MZ B and iNK T cells, which are potent regulators of the immune response [96]. Moreover, *Turicibacter* was the only genus positively affected by the reduction in CD8^+^ T cells. Thus, our method identified a taxon that is potentially directly linked to IBD via the modulation of the immune system.

Perhaps the most unexpected finding was our detection of *A. segnis* and *Sutterella* as the only species and genus increased in disease compared to 26 positive associations detected by the previous analysis [25]. All other associations were classified as indirect even though they often corresponded to much more significant changes in abundance between IBD and control groups. Thus, our results indicate that expansion of many taxa including opportunistic pathogens is driven by their interactions with the core IBD network shown in Fig 3. One possibility is that the dysbiosis of the symbiotic microbiota makes it less competitive against other bacteria and opens up niches that can be colonized by opportunistic pathogens. The other, less explored possibility, is that commensal microbiota can not only protect from pathogens, but also facilitate their invasion, a phenomenon that has been recently demonstrated in bees [97].

Little is known about the specific roles that *A. segnis* and *Sutterella* play in IBD, and more generally in gut health. *Aggregatibacter* is a common member of the oral microbiota that thrives in local infections such as periodontal disease and bacterial vaginosis [98–100]. The high abundance of *A. segnis* is also associated with an increased risk of IBD recurrence [101]. *Sutterella*, on the other hand lacks overt pathogenicity, and MWAS produced inconsistent findings [102–108] on its involvement in IBD. Some studies reported that *Sutterella* is increased in patients with good outcomes [21, 105] while other studies found positive or no association between *Sutterella* and IBD [25, 103, 106–108]. Experimental investigations showed that *Sutterella* lacks many pathogenic properties; in particular, it does not induce a strong immune-response and has only moderate ability to adhere to mucus [107, 108]. Further, *Sutterella* strains from IBD and control patients showed no phenotypic differences in metabolomic, proteomic, and immune response assays [108]. Nevertheless, *Sutterella* is strongly associated with worse behavioral scores in children with autism spectrum disorder and Down syndrome [19, 20, 109]. Therefore, the direct link between *Sutterella* and IBD could involve the gut-brain axis.

In summary, we found a small number of taxa can explain extensive dysbiosis in IBD and accurately predict disease status. Directly associated taxa have strains with dramatically different abilities to trigger colitis and are specifically targeted by the immune system of patients and animals with IBD [12]. Previous studies of these taxa point to facilitated colonization by pathogens, butyrate production, immunomodulation, bile metabolism, and the gut-brain axis as the primary factors in the etiology of IBD.

Many disorders are accompanied by substantial changes in host microbiota, but our work shows that only a small subset of these changes could be directly related to the disease. Similarly, only a handful of taxa could drive the dynamics of the ecosystem-level changes in the environment. To untangle the complexity of such dysbioses, it is important to account for microbial interactions using mechanistic or statistical methods. Direct association analysis proposed in this paper is a simple statistical approach based on the principle of maximum entropy. DAA can be applied to any microbiome data set that is sufficiently large to infer interspecific interactions.

## Methods

The data used in this study was obtained from Ref. [21], which reported changes in the microbiome of newly-diagnosed, treatment-naive children with IBD compared to controls. This data was recently analyzed in Ref. [25], and we followed all the statistical procedures adopted in that study to enable direct comparison of the results. Specifically, we used a permutation test on mean log-transformed abundances to determine the statistical significance of an association.

To fit the maximum entropy model to the data, we first computed the mean log-abundance for each genus *m*_*i*_ and the covariance in the log-transformed abundances *C*_*ij*_. The interaction matrix was computed as *J* = *C*^−1^ by performing singular value decomposition [110] and removing all singular values that were comparable to the amount of noise present in the data. The host effects were computed as *h* = *Jm*. See S1 Text for further details.

All computation was carried out in Python environment. We used scikit-learn 0.15.2 [111] for hierarchical clustering and to build the supervised classifiers used in Fig 4B of the main text and S8 Fig. The variance in the accuracy of classification was evaluated through 5-fold stratified cross-validation with 100 random partitions of the data into the training and validation sets. For all findings, statistical significance was evaluated with Fisher’s exact test (permutation test) with 10^6^ permutations. False discovery rate was controlled to be below 5% following Benjamini-Hochberg procedure [60].

For sparse logistic regression, we confirmed that the penalty parameter was in the range where the results are insensitive to its specific value. The features selected by this classifier in Fig 4 are as follows: *Erysipelotrichales*, *Pasteurellales*, *Turicibacterales* (also significant in DAA), and *Enterobacteriales* (not significant in DAA) at the order level; *Clostridiaceae* and *Pasteurellaceae* (also significant in DAA) and *Enterobacteriaceae* and *Erysipelotrichaceae* (not significant in DAA) at the family level; *Roseburia* (also significant in DAA) and *Dialister*, *Aggregatibacter*, and *Haemophilus* (not significant in DAA) at the genus level; and *B. adolescentis*, *F. prausnitzii*, and *E. dolichum* (also significant in DAA) and *Prevotella copri* and *Haemophilus parainfluenzae* (not significant in DAA) at the species level. In total, both DAA and the sparse logistic regression relied on 17 features with 9 of them being the same. Thus, DAA identified many features that were also selected by the machine learning algorithm for their predictive value. At the same time, the results of DAA and the sparse logistic regression were not exactly the same and, therefore, could be complementary to each other.

## Supporting information

S1 Text Supplementary text and tables. Derivation of mathematical model of community composition and inference of model parameters, discussion of assumptions and limitations of DAA.
(PDF)

S1 Fig Microbial abundances follow the log-normal distribution. The histograms show probability distributions of the relative log-abundance for the species and genera detected by DAA (summarized in Fig 3). The best fit of a Gaussian distribution is shown in green.
(TIF)

S2 Fig Pairwise interactions are sufficient to explain the patterns of microbial co-occurence. The parameters in our maximum entropy model were chosen to fit only the first and the second moments of the multivariate distribution of microbial abundances. Nevertheless, the model captures most of the higher-order correlations in the data suggesting pairwise interactions are sufficient to accurately describe the patterns of microbial co-occurences. (**A**) For each choice of three genera, the third order moment was computed by averaging the product of the log-abundances over all the samples in the IBD data (“observed”) or from Eq. (17) (“predicted”), which states the predictions of the maximum entropy model. The plot shows excellent agreement between the two quantities. (**B**) For each choice of three genera (“index”), we plot the third-order central moment computed from the IBD data (“observed”) and from an equally-sized sample drawn from our maximum entropy model (“Gaussian distribution”). The latter quantifies the expected deviations between the observations and predictions due to the finite size of the sample. (**C**) Same as (A), but for the fourth-order central moment. The expected level of noise is quantified via a sample from the maximum entropy model that obeys Eq. (17) exactly in the limit of infinite sample size. The correlation coefficient between “observed” and “predicted” values from this sample sets the upper bound on the expected correlation coefficient in IBD data.
(TIF)

S3 Fig Microbial interactions are only weakly affected by host phenotype. To determine whether Crohn’s disease drastically alters the pattern of microbial interactions, we computed and compared the covariance matrixes *C*^CD^ and *C*^control^ for CD and control groups respectively. The results of this calculation for IBD data are shown in blue. Each dot corresponds to a matrix element of *C*_*ij*_, which is the covariance between the log-abundances of genera i and j. The *x*-coordinate is the covariance computed in the control group and the *y*-coordinate is the covariance computed in the CD group. To estimate the expected level of noise, we carried out the same analysis on two random partitions of the data that contain both controls and subjects with CD (shown in magenta). Since the groups are drawn from the same distribution, their covariance matrices must be identical on average. The spread of the magenta data points, therefore, sets the upper limit on the correlation coefficient between *C*^CD^ and *C*^control^. We note, however, that this upper bound is unlikely to be reached for IBD data because some taxa have different noise levels in CD and control groups: eg. the taxa depleted in CD have a low abundance in this group and, therefore, higher error in the estimates of the correlation coefficients with other taxa. Overall, both IBD and partitioned data lie close to the diagonal and exhibit similar levels of variation. Thus, using the same covariance matrix for both CD and control groups is a reasonable first approximation. This approximation is valuable because it reduces the uncertainty in *C*_*ij*_ by allowing us to use the entire data to compute covariances and because it improves the stability of DAA to errors in *C* (see S12 Fig).
(TIF)

S4 Fig Taxa directly associated with Crohn’s disease. Note that the Green Genes database [112] used in QIIME [113] places Turicibacter under Erysipelotrichales and has a unique order of Turicibacterales. This apparent inconsistency may reflect insufficient understanding of Turicibacter phylogeny. The effect sizes and statistical significance are summarized and results for DAA and conventional MWAS are compared in S1 Text.
(TIF)

S5 Fig Comparison between correlations and direct interactions. The matrix of microbial interactions *J* is shown in (**A**) and the correlation matrix *C* is shown in (**B**), which is the same as Fig 1B of the main text. Both matrices are inferred from the IBD data set. Note that *J* is sparser than *C*. For greater clarity, the matrices are hierarchically clustered; therefore, the order of species in (A) and (B) is not the same.
(TIF)

S6 Fig Comparison of networks inferred by Pearson correlation, SparCC, and DAA at the genus level. Three networks quantifying microbial co-occurrence or interactions have been inferred: one based on the Pearson correlation coefficient between log-abundances (which is closely related to the covariance matrix *C*), one using SparCC package from Ref. [56] that attempts to reduce compositional bias, and one based on the direct interactions *J* from DAA. In each network, we kept only links that were statistically different from 0 under a permutation test with 5% false discovery rate. The panels display Venn diagrams showing unique and overlapping links in these networks. All links are included in (**A**), and the comparison is done irrespective of the sign of the link, i.e. agreement is reported even if one method reports a positive link and another method reports a negative link. In contrast, (**B**) and (**C**) show only positive and negative links respectively. Three conclusions can be drawn from these comparisons. First, the high overlap between SparCC and Pearson networks shows that log-transforms have largely accounted for the compositional bias. Second, all three methods agree on a large number of links suggesting that all methods are sensitive to some strong interactions. Third, DAA reports fewer links and identifies a few links not detected by other methods. This reflect the different nature of DAA links. While both Pearson correlation and SparCC infer correlation, which could be either direct or indirect (i.e. induced; see main text). DAA removes indirect correlations, thus reducing the total number of links, but also reveals pairwise interactions that could have been masked by strong correlations with a third species.
(TIF)

S7 Fig The network based on the correlation coefficient between log-transformed abundances. We plotted the correlation-based network for the species detected by DAA. Note the similarities and differences with the interaction network shown in Fig 3 of the main text. Only the links with the correlation coefficient greater than 0.27 or lower than -0.15 are shown, and all links are statistically significant (*q* < 0.05). All correlation coefficients and direct interactions are summarized in S1 Text for the genera and species detected by DAA.
(TIF)

S8 Fig Direct associations retain full diagnostic power. The same as Fig 4B of the main text, but for two other classifiers: random forest [64, 65] in (**A**) and support vector machine [66] in (**B**).
(TIF)

S9 Fig DAA detects all directly associated taxa in synthetic data, provided the sample size is sufficiently large. The same as Fig 2A in the main text, but with the *x*-axis extended to larger sample sizes. Note that DAA recovers all 6 directly associated taxa when the sample size is greater than about 1200.
(TIF)

S10 Fig Compositional bias has a negligible effect on DAA performance. All panels are the same as Fig 2C in the main text, but with different normalization of the data prior to the analysis. (**A**) No normalization: the analysis is done on the counts from the OTU table, which do not add up to a constant number. (**B**) Total-sum scaling: The counts are converted into relative abundances by dividing by the total number of counts (reads) per sample. This plot is the same as Fig 2C. (**C**) Centered-log ratio: First log-abundances were computed from unnormalized counts with a pseudocount of 1. Then, the mean log-abundances of the taxa was computed by averaging over the samples. Finally, the mean-log abundance of every taxon was subtracted from the log-abundances of this taxon in all samples. This procedure corresponds to normalizing by the geometric mean of the counts because it ensures that the mean log-abundance of a taxon is zero [55]. (**D**) Cumulative sum scaling: A normalization scheme proposed specifically for microbiome analyses was implemented following Ref. [114]. The results of the analyses in (A)-(D) are very similar suggesting that compositional bias does not lead to major artifacts. In particular, the number of associations in (A) grows at the same rate with the sample size as in (B)-(D). This would not be the case if the compositional bias was strong because spurious associations due to normalization would lead to a greater number of detected taxa. Thus, we conclude that interspecific interactions rather than compositional effects are the primary source of spurious associations.
(TIF)

S11 Fig The inference of the eigenvalues of the covariance matrix is robust to variation in sample size and bootstrapping. We repeatedly subsampled the IBD data set to half of its size and computed the eigenvalues of the covariance matrix *C*. The means and standard deviations from this bootstrap procedure are shown in green, and the eigenvalue inferred from the entire data are shown in black. The agreement between the different sample sizes and the small variation due to subsampling indicate that the spectral properties of *C* can be inferred quite accurately.
(TIF)

S12 Fig Results of DAA are robust to variation in sample size and bootstrapping. Similar to S11 Fig, we repeatedly subsampled the IBD data set to half of its size and carried out DAA on each of the subsamples. (**A**) shows that there is a modest variation in inferred *h*. To a large extent, this variation is driven by the uncertainty in *C* and its inverse *J*. (**B**) shows a much smaller variation in Δ*h* between control and CD groups (green symbols). The noise is reduced because, even though *C* changes from subsample to subsample, the same *C* is used to infer *h* for control and disease groups. Therefore, the variability in *C* has a much weaker effect on Δ*h*. For comparison, we also show Δ*h* obtained by bootstrapping the entire data set without preserving the diagnosis labels (black symbols). These data show the expected distribution of Δ*h* under the null hypothesis of no associations. For genera detected by DAA, the black and the green error bars do not overlap suggesting that the results of DAA are not affected by the uncertainty in *C* and are robust to variation in sample size and bootstrapping.
(TIF)

S13 Fig Results of DAA are not significantly affected by compositional effects. The quantity Δ*h* between control and CD groups is the test statistic used to infer direct associations, and the variation of Δ*h* due to sampling shows whether the statistical analysis is robust to small changes in the data set. To quantify these variations in Δ*h*, we consider a sample drawn from the maximum entropy model fitted to the IBD data set and define two *δ*Δ*h*: one between normalized and not normalized sample and the other between the not normalized sample and the values of *h* in the maximum entropy model. The first *δ*Δ*h* quantifies the variability due to normalization, while the second *δ*Δ*h* quantifies the variability due to sampling. The plot shows the distribution of the absolute values of the difference between the absolute values of these *δ*Δ*h* across genera for three normalization schemes: total-sum scaling (TSS), centered-log ratio (CLR) and cumulative sum scaling (CSS). The absolute Δ*h* values of significant taxa in IBD RISK data (red rectangles) lie well outside of the distributions shown.
(TIF)

S14 Fig Spurious associations in synthetic data with small and large effect sizes. The same analysis as in Fig 2A and 2B of the main text, but for synthetic data with smaller (A, B, C) and larger (D, E, F) effect sizes. (**A**) and (**D**) show the number of associations detected by traditional MWAS and DAA. (**B**) and (**E**) show the median effect sizes (median fold change) for the taxa detected by conventional MWAS. (**C**) and (**E**) show the effect sizes in both *h* and *l* for the taxa detected by DAA. The effect size for *h* was quantified by the relative difference in *h* between cases and controls. The effect size for *l* was quantified as in (B) and (E). Overall the results are similar to those in Fig 2. In addition, (A) and (B) show that DAA can recover all directly associated taxa given a large number of samples without any false positives. For sample sizes exceeding 5000, DAA starts to detect indirect associations due to compositional effects.
(TIF)

S15 Fig Sensitivity of DAA to eigenvalue threshold λ_min_. Large λ_min_ retains only a few eigenvalues and imposes an artificially strong correlation structure on the data. As a result, DAA detects a large number of associations because it cannot distinguish direct from indirect effects. The performance of DAA improves as more eigenvalues are included and reaches a plateau. The dashed lines show the number of eigenvalues included for λ_min_ = 0.01 used throughout our analysis. The insets show the eigenvalues of Λ in decreasing order. The four panels show the results for different taxonomic levels: from species to order.
(TIF)
