# Supplementary material for: Interactions between species introduce spurious associations in microbiome studies
Source: PLoS Comput Biol. 2018 Jan 16;14(1):e1005939. doi: 10.1371/journal.pcbi.1005939 (PMC5786326; doi:10.1371/journal.pcbi.1005939)
Supplement: S1 Text — Derivation of mathematical model of community composition and inference of model parameters, discussion of assumptions and limitations of DAA. (PDF) [file pcbi.1005939.s001.pdf]

Supplementary information for “Interactions between species introduce spurious associations in microbiome studies”

Model of community composition

Here we describe a mathematical model of community composition, that we use to correct for microbial interactions in microbiome-wide association studies.

Log-transformation of abundances
The environment within a host is constantly changing due to variations in diet, immune response, phage activity and other factors. As a result, microbial growth rates should be highly variable and produce multiplicative fluctuations in the community composition, which are better captured on logarithmic rather than on linear scale. Indeed, the abundances of many gut species follow a log-normal distribution (Fig. S1), and recent work shows that a log-transformation of abundances increases the power and quality of microbiome studies [25]. Therefore, we chose to carry out all of the analysis and modeling on natural logarithms of relative abundances computed with a pseudocount of one read. For simplicity, we refer to these quantities as abundances in the following and denote them as l_i with the subscript identifying the species under consideration.

Maximum entropy models
Microbiota composition is highly variable among people in both health and disease [25] and needs to be described via a multivariate probability distribution $P(\{l_i\})$. The amount of data in a large microbiome-wide association study, however, is sufficient to reliably determine only the first and second moments of $P(\{l_i\})$. This situation is common in the analysis of biological data and has been successfully managed with the use of maximum entropy distributions [38]. These distributions are chosen to be as random as possible under the constraints imposed by the first and second moments. Maximum entropy models introduce the least amount of bias and reflect the tendency of natural systems to maximize their entropy. In other contexts, these models have successfully described the dynamics of neurons [50], forests [51], and flocks [52], and even predicted protein structure [53] and function [54]. In the context of microbiomes, a recent work derived a maximum entropy distribution for microbial abundances using the principle of maximum diversity [55].

Let us denote abundance means and covariances computed from the data by the vector m and matrix C respectively. The constraints on the maximum entropy distribution are then expressed as

$$\begin{aligned} \langle l_i \rangle &= m_i \\ \langle l_i l_j \rangle - \langle l_i \rangle \langle l_j \rangle &= C_{ij} \end{aligned} \tag{1}$$

and the maximum entropy distribution takes the following form

$$P(\{l_i\}) = \frac{1}{Z} e^{\sum_i h_i l_i + \frac{1}{2} \sum_{ij} J_{ij} l_i l_j} \tag{2}$$

which is similar to the Ising model of statistical physics, but with continuous rather than discrete degrees of freedom. The variables h_i and J_{ij} arise as Lagrange multipliers for the first and second

moment constraints during entropy maximization. In statistical physics, they describe local magnetic fields that align spins l_i and interactions between spins l_i and l_j. The constant Z, known as the partition function, ensures that the distribution is normalized:

$$Z = \int \prod_i dl_i e^{\sum_i h_i l_i + \frac{1}{2}\sum_{ij} J_{ij} l_i l_j} \tag{3}$$

Note that Z is a multi-dimensional Gaussian integral.

Host effects vs. species interactions
To interpret this maximum entropy distribution in terms of biologically relevant factors such as microbial interactions and properties of the host, we can rewrite equation (2) as follows

$$P(\{l_i\}) = \frac{1}{Z} e^{\sum_i H_i l_i} \tag{4}$$

where

$$H_i = h_i + \frac{1}{2}\sum_j J_{ij} l_j \tag{5}$$

describe the quality of the local environment for species i: the higher H_i, the more abundant the species. The quality of the environment can be decomposed into external variables such as temperature or metabolite concentrations V_α and the species' response to these variables $R_{i\alpha}$ as

$$H_i = \sum_\alpha R_{i\alpha} V_\alpha \tag{6}$$

We can further decompose the external variables V_α into host factors V_α^h and influences of other species, e.g., due to metabolite secretion or production of antibiotics:

$$V_\alpha = V_\alpha^h + \sum_j P_{\alpha j} l_j \tag{7}$$

where $P_{\alpha j}$ describes the influence of microbe j on variable α.

Upon combining equations (6) and (7), we can express H_i as

$$H_i = \sum_\alpha R_{i\alpha} V_\alpha^h + \sum_{\alpha j} R_{i\alpha} P_{\alpha j} l_j \tag{8}$$

Comparison of this equation to equation (5) shows that we can identify $h_i = \sum_\alpha R_{i\alpha}V_\alpha$ with the direct effects of the host and $J_{ij} = 2\sum_\alpha R_{i\alpha}P_{\alpha j}$ with the interactions among the microbes.

Inference of model parameters

Here we describe the procedure of learning the parameters of the maximum entropy model from the data. Our approach closely follows that of Refs. [38], [53] and [54].

Relating h and J to m and C
To infer model parameters h_i and J_{ij}, we need to relate them to empirical observations such as the means and covariances of the abundances. These relationships can be conveniently obtained from the derivatives of the partition function, which is the standard approach in statistical physics. Indeed, the mean abundances can be expressed as

$$\langle l_k \rangle = \frac{1}{Z}\int \prod_i dl_i e^{\sum_i h_i l_i + \frac{1}{2}\sum_{ij} J_{ij} l_i l_j} l_k = \frac{\partial \ln Z}{\partial h_k}. \tag{9}$$

A similar relationship holds for the covariance matrix:

$$\langle l_i l_j \rangle - \langle l_i \rangle \langle l_j \rangle = \frac{\partial^2 \ln Z}{\partial h_i \partial h_j} \tag{10}$$

To complete the calculation, we need to compute the partition function defined by equation (3). The result reads

$$Z = \frac{1}{\sqrt{\det(J/2\pi)}} e^{\frac{1}{2} h^T J^{-1} h} \tag{11}$$

where symbols without indexes are treated as vectors or matrices.

From equation (11), we immediately find that

$$\begin{aligned} m &= J^{-1}h \\ C &= J^{-1} \end{aligned} \tag{12}$$

which can be inverted to obtain

$$\begin{aligned} h &= C^{-1}m \\ J &= C^{-1} \end{aligned} \tag{13}$$

Inverting the covariance matrix
It is clear from equation (13) that the key step in obtaining the model parameters is the inversion

of the covariance matrix. However, this matrix is likely to be degenerate or ill-conditioned because of the insufficient amount of data or very strong correlations between microbial abundances. To overcome this difficulty, we computed a pseudoinverse of C as described in the following sections. Briefly, we used singular value decomposition [114] of C in terms of two orthogonal matrices U and V (since C is symmetric, $U = V$) and a diagonal matrix Λ:

$$C = U\Lambda V^T \tag{14}$$

Some diagonal elements of Λ were small and comparable to the levels of noise (or uncertainty), so we set the corresponding elements of Λ^{-1} to zero. Specifically, Λ^{-1}_{kk} was set to zero for all k such that $\Lambda_{kk} < \lambda_{\min}$, where λ_{min} was a predetermined threshold. A regular inverse ($\Lambda^{-1}_{kk} = 1/\Lambda_{kk}$) was used for the rest of the elements. The choice of the threshold and the robustness of the results to the variation in $\lambda_{\min}$ are discussed in the section on data analysis. This procedure ensured that we do not infer large changes in host fields h due to fluctuations in the estimate of $\langle l \rangle$. The inverse of C was then computed as $C^{-1} = V\Lambda^{-1}U^T$, where we used the fact that the inverse of an orthogonal matrix is its transpose.

Origin of spurious associations and Direct Associations Analysis

Microbial interactions introduce spurious associations

In microbiome-wide association studies, we are typically interested in the changes in microbial abundances Δm between two groups of subjects. From equation (12), we can relate Δm to the changes in the phenotype of the host Δh:

$$\Delta m = C\Delta h \tag{15}$$

This formula clearly illustrates the origin of spurious associations. Imagine that there is a small number of species directly linked to host phenotype, i.e. Δh is a sparse vector. Because C is a dense matrix (see Fig. 1b in the main text), equation (15) predicts that Δm is dense, i.e. the abundances of most species are affected. The sizes of these effects are variable and depend on the magnitude of the off-diagonal elements of C. Except for the strongly interacting species, the largest changes in m are likely to mirror the largest changes in h and result in significant associations. In large samples, however, smaller effects become detectable that could either reflect small direct effects or the secondary, indirect effects due to microbial interactions. As a result, the number of associations grows with the sample size, and the relationship between associated species and host phenotype becomes obscured. Fig. 2 in the main text presents evidence for a large number of spurious associations in both synthetic and real data.

Removing indirect associations

Equation (15) offers a straightforward way to correct for microbial interactions and separate direct from indirect associations. Indeed, for each species, we can compute the corresponding change in the host field as

$$\Delta h_i = \sum_j \left(C^{-1}\right)_{ij} \Delta m_j \tag{16}$$

The statistical significance of this change can be determined via the permutation test followed by the Benjamini-Hochberg procedure to correct for multiple hypothesis testing [61].

Assumptions and limitations of DAA

Pairwise interactions are sufficient
So far, we have considered only pairwise interactions between the taxa. This is a common assumption in maximum entropy models, which reflects the need for very large data sets in which higher-order interactions can be reliably inferred [38, 50–54]. While fitting higher-order interactions is impractical, we can nevertheless test whether they make a significant contribution to the patterns of co-occurrence observed in IBD data. To this purpose, we computed third and fourth order moments of microbial abundances in IBD data and compared them to the corresponding moments predicted by our maximum entropy model. This is a meaningful test because only the first and second moments were used to fit the model to the data.

The predictions of our model follow from the properties of the multivariate Gaussian distribution and can be summarized as follows:

$$\begin{aligned}
&\langle l_i l_j l_k \rangle = m_i m_j m_k + m_i C_{jk} + m_j C_{ik} + m_k C_{ij} \\
&\langle (l_i - \langle l_i \rangle)(l_j - \langle l_j \rangle)(l_k - \langle l_k \rangle) \rangle = 0 \\
&\langle (l_i - \langle l_i \rangle)(l_j - \langle l_j \rangle)(l_k - \langle l_k \rangle)(l_m - \langle l_m \rangle) \rangle = C_{ij} C_{km} + C_{im} C_{jk} + C_{ik} C_{jm}
\end{aligned} \tag{17}$$

The model predicts that the third central moments vanish, and indeed the corresponding values in the data are close to zero (Fig. S2). The observed deviation is consistent with the level of noise seen in a random Gaussian sample drawn from the maximum entropy distribution; the size of the sample equaled that of the IBD data. Further, the predictions for the non-central moments are highly correlated with the moments observed in IBD data (Fig. S2) with Pearson's r equal to 1 and 0.81 for third and fourth moments respectively. The deviations of r from 1 are largely due to the uncertainty in the values of the observed moments. Indeed, we obtained $r = 1$ and $r = 0.88$ for the correlation between predicted and observed third and fourth order moments for the random sample drawn from our maximum entropy distribution. Since the higher moments of the maximum entropy distribution satisfy Eq. (17) exactly, the observed values of r set the upper bound on the correlation coefficient that can be obtained given the sample size in the IBD data set.

Host phenotype affects h, but not J
An important assumption behind Eq. (16) is that the interspecific interactions are not affected by host phenotype, i.e. C and J are the same for control and disease groups. Deviations from this assumption are certainly possible, but they represent higher order effects, which are absent in a simple linear-response model of microbial communities given by Eq. (8). Moreover, current sample sizes are insufficient to accurately infer and compare the covariance matrices for each of the groups. Association tests between microbial interactions and host phenotype are further complicated by the large number of interspecific interactions, which leads to a severe reduction in statistical power.

Therefore, we did not attempt to identify specific interactions that are affected by IBD; instead, we assessed the overall similarity between the covariance matrices C^{CD} and C^{control} computed for patients with and without Crohn's disease (Fig. S3). We found that the plot of the matrix elements of C^{CD} vs. C^{control} clustered around the diagonal with the coefficient of linear regression equal to 0.96, suggesting that the structure of correlations is similar for the two phenotypic groups. The spectral properties of the matrices are also similar.

To perform a more quantitative comparison we also computed the Pearson correlation coefficient between the matrix elements of C^{CD} vs. C^{control} ($r = 0.7$). However, interpreting the value of the correlation coefficient is non-trivial because it is very sensitive to the noise in the data and the uncertainty in the individual matrix elements is high, especially for taxa with low abundance. One way to estimate the expected level of noise is to compare the observed correlation coefficient to the correlation coefficient for two subsamples of the shuffled data drawn without preserving the diagnosis labels, but of the same size as the CD and control groups. This coefficient must equal 1 in the limit of infinitely large data, so it sets the upper limit on r that can be observed between C computed for CD and control groups, even when there are no differences in the interactions. We note, however, that this upper bound is unlikely to be reached for IBD data because some taxa have different noise levels in CD and control groups. Indeed, the taxa depleted in CD have a low abundance in this group and, therefore, higher error in the estimates of the correlation coefficients with other taxa. We found that the correlation coefficient r between two random subsets was about 0.9, suggesting that high level of noise is the likely explanation for the spread of the data away from the diagonal in Fig. S3.

Robustness of inference to the uncertainties in the covariance matrix

Since the sample size in the IBD data set is not sufficient to infer every element of the covariance matrix accurately, it is important to determine how the uncertainty in C affects DAA results. To this end, we repeatedly subsampled the IBD data set to half of its size and examined the variation in the gross properties of C and changes in h and Δh. Fig. S11 shows that the eigenvalues of C are extremely robust and are virtually unaffected by the subsampling of the data. Similarly, there is only small variation in the values of Δh between control and CD groups (Fig. S12). For genera detected by DAA, the values of Δh together their error bars due to subsampling are well outside the region where Δh are expected to lie under the null hypothesis of no association between the genus and Crohn's disease.

Compositional effects

Microbial abundances are usually normalized by the total number of reads in the sample to eliminate the noise introduced during sample preparation, for example, at DNA extraction and amplification steps. Other normalization schemes are also used because they could be advantageous for certain data or analyses [55, 59, 60]. Any normalization eliminates one dimension of the data and thereby creates compositional biases that complicate the interpretation of the results [56–58]. For example, the *relative* abundance of a microbe could change simply due to the change in the abundance of other members in the community; such a possibility makes it difficult to unambiguously determine whether this microbe is associated with host phenotype. While it is impossible to fully eliminate compositional biases, their effects could be minimized. In this section, we show that the procedure that we adopted to compute C^{-1} achieves such minimization for a particular choice of the normalization scheme. We also discuss how DAA can be generalized for an arbitrary normalization scheme and show that the same results are obtained with and without the normalization of the data prior to the analysis. Overall, we conclude that compositional biases do not affect the performance of DAA for diverse microbial communities such as the gut and sample size less than about 5000. The

application of DAA to data with strong compositional effects would require the modifications that we outline below.

In this section, we use l_i to denote the log-transformed abundance of microbe i regardless of the normalization scheme. The log-transformation is an important step in the analysis of compositional data because it reduces the degree of compositional biases [55–60]. Any normalization of the data imposes a constraint on l_i, which can be stated as follows

$$F(\{l_i\}) = 0 \tag{18}$$

The normalization that we used so far, known as total-sum scaling [59], corresponds to

$$F(\{l_i\}) = -1 + \sum_i e^{l_i} \tag{19}$$

while another popular normalization scheme, known as centered-log ratio, corresponds to

$$F(\{l_i\}) = \sum_i l_i \tag{20}$$

The requirement that $F(\{l_i\}) = 0$ changes the maximum entropy distribution to

$$P(\{l_i\}) = \delta(F(\{l_i\}))\frac{1}{Z_F}e^{\sum_i h_i l_i + \frac{1}{2}\sum_{ij} J_{ij} l_i l_j} \tag{21}$$

where $\delta(\cdot)$ is the Dirac delta function, and the subscript on Z indicates that the normalization constant depends on the choice of F. It is easy to show the origin of Eq. (21) by replacing the hard constraint in Eq. (18) by a soft constraint on the moments of $P(\{l_i\})$. Hard constraints are rarely included in the maximum entropy models while the inclusion of soft constraints is the standard practice. Specifically, we can replace Eq. (18) by

$$\begin{aligned} \langle F(\{l_i\})\rangle &= 0 \\ \langle F^2(\{l_i\})\rangle &= \theta^2 \end{aligned} \tag{22}$$

which is equivalent to Eq. (18) in the limit of $\theta \to 0$. The maximum entropy distribution satisfying Eq. (22) reads

$$P(\{l_i\}) = \frac{1}{Z_\theta}e^{\sum_i h_i l_i + \frac{1}{2}\sum_{ij} J_{ij} l_i l_j}e^{-\frac{F^2(\{l_i\})}{2\theta^2}} \tag{23}$$

which reduces to Eq. (21) as $\theta \to 0$.

The delta function or the new $\theta-$dependent term changes the maximum entropy distribution, and Eq. (12) no longer hold for a general choice of $F(\{l_i\})$. Instead, one has to compute the first and second order moment of the distribution given by Eq. (21) or Eq. (23) and fit them to the means and covariances observed in the data. This procedure, however, cannot uniquely determine h_i and J_{ij} because these parameters are no longer independent. Indeed, the condition that $\langle F^2(\{l_i\})\rangle = 0$ imposes a constraint on the values that h_i and J_{ij} can take. This constraint is the consequence of the fact that normalization destroys one dimension of the data. The maximum entropy model "inherits" this property, so any change in h_i could in part be due to the compositional bias.

Accounting for compositional affects for an arbitrary F is nontrivial and is hardly justified given the weak compositional effects in the IBD data set. The analysis is, however, quite straightforward for F given by Eq. (20), which corresponds to the normalization by the geometric rather than arithmetic mean of microbial abundances. We now use this choice of F to illustrate the general principles outlined above and to demonstrate that our implementation of DAA already accounts for the compositional bias for this normalization scheme.

For F given by Eq. (20), the soft constraint introduces a factor that keeps $P(\{l_i\})$ a multivariate Gaussian distribution. Therefore, Eq. (23) is equivalent to our original model given by Eq. (2) with J replaced by $J^{(\theta)}$ defined as

$$J_{ij}^{(\theta)} = -\frac{1}{\theta^2} + J_{ij} \qquad (24)$$

In the matrix notation, this definition takes the following form

$$J^{(\theta)} = -\frac{1}{\theta^2}E + J \qquad (25)$$

where E is the matrix with all elements equal to 1.

Equations (12) then continue to hold and can be used to infer $h^{(\theta)}$ and $J^{(\theta)}$. As $\theta \to 0$, $J^{(\theta)} \to J$ in the subspace of $\sum_i l_i = 0$, i.e. except in the direction of $(1, 1, ..., 1, 1)^T$, which becomes the eigenvector of $J^{(\theta)}$ with a very large eigenvalue. This direction is also an eigenvector of C, and the corresponding eigenvalue tends to zero. Thus, compositional effects render C degenerate. Strong microbial interactions can have the same effect, and we indeed found a few vanishingly small eigenvalues of C. The variation in the data along the degenerate directions is eliminated when we calculate C^{-1} using the singular value decomposition [114] as explained in the corresponding section above.

This procedure does not artificially exclude taxa from the analysis. For example, if two microbes are perfectly correlated with each other, DAA reports both as significant associations if their abundances vary between health and disease. Since DAA dramatically reduces the number of associations compared to conventional MWAS, we conclude that most of the spurious associations are driven by microbial interactions rather than the compositional bias. Further, the small number of associations found by DAA with quite different relative abundances makes it unlikely that they arise due to compositional effects.

Nevertheless, the maximum entropy model does "inherit" a constraint on the parameters from the compositional nature of the data. For $F(\{l_i\}) = \sum_i l_i$, it is easy to see that $\sum_i h_i$ cannot be uniquely determined from the data. Indeed, adding the same constant to every h_i changes the exponent in the expression for $P(\{l_i\})$ by a factor proportional to $\sum_i l_i$, which must vanish due to the delta function. One can then choose an arbitrary value for $\sum_i h_i$, say set it to zero. This condition reflects the residual compositional bias left in the maximum entropy model. Similarly, due to the compositional constraint on l_i, the constraint on h_i can force h_i to be different for all taxa, even if only one of them is directly affected by the host phenotype. The effect of the constraint, however, should scale as one over the number of the taxa that fluctuate independently. For a diverse ecosystem such as the gut, the effect of the compositional bias should, therefore, be small and detectable only with very large sample sizes. In the synthetic data, we start seeing the compositional effects at about 5000 samples which is 10 times the number of samples in the IBD data set; see Fig. S14.

To test for compositional biases in the results of DAA, we analyzed the IBD data set with several widely-used normalization schemes [55, 59], including total-sum scaling, centered-log ratio, cumulative sum scaling, and no normalization at all (Figs. S10 and S13). All analyses identified about the same number of associations (and the same taxa) using either traditional MWAS or DAA. Finally, we note that our synthetic data has the same amount of compositional bias as in the IBD data. For both data sets, the top 10 most abundant taxa account for 80 % of the reads, and we normalized the synthetic data by the total number of reads in the sample prior to performing DAA.

Generation of synthetic data

Here, we describe how we generated the synthetic data shown in Fig. 2A of the main text. This data was generated to evaluate the likelihood of spurious associations in MWAS. We introduced a known number of direct associations, but ensured that all other properties of the data correspond to that of the human gut microbiota.

The data for the control group were directly subsampled from the IBD data set. To generate the data for the disease group, we first inferred the covariance matrix using the entire data set and the mean abundances using just the control group. Then, equation (12) was used to compute h. These values of h described normal microbial abundances in subjects without IBD. To introduce a difference between cases and controls, we modified the values of h for 6 randomly chosen species by 10% - 40%; these are typical changes in h identified by DAA. Finally, we computed the expected microbial abundance using equation (12) and then sampled from a multivariate Gaussian distribution with these means and the covariance matrix defined above.

We also tested that our conclusions hold for other diseases with potentially different effect sizes. Specifically, we repeated the analysis in Fig. 2A for two other synthetic data sets: one with smaller and one with larger effect sizes. The results are qualitatively similar to what we reported in the main text and are shown in Fig. S14. The values of the effect sizes are given in Tab. S2.

Data analysis

For correlation analysis, we used Pearson correlation coefficient for log-transformed abundances.

For logistic regression classifier, we used L1 penalty to ensure sparseness and generalizability. In

all classifiers default parameters were used in scikit-learn version 0.17.2.

For hierarchical clustering of the correlation matrix, we used the Nearest Point Algorithm method of the linkage function in scipy with a correlation distance metric.

Threshold for matrix inversion

For our analysis of the IBD and synthetic data sets we set $\lambda_{\min}$ to 0.01. To test whether our results are robust to the value of the threshold, we varied the number of eigenvalues of Λ^{-1} not set to zero; see Fig. S15. When only a few eigenvalues where included, DAA detected a large number of associations because many taxa were perfectly correlated, and it was impossible to distinguish direct from indirect associations. As the number of included eigenvalues increased, the performance of DAA improved and reached a plateau. In this plateau region, the results were largely insensitive to the value of the threshold used. Our choice of the theshold corresponded to this plateau region. At all taxonomic levels, we found one or two almost zero eigenvalues that were below λ_{min} (Fig. S11); all other eigenvalues were included in the analysis.

Computer code

We include here the link to computer code that loads the data and outputs all figures and tables: https://github.com/rajitam/DAA-figures-and-tables

Fig. S1. Microbial abundances follow the log-normal distribution. The histograms show probability distributions of the relative log-abundance for the species and genera detected by DAA (summarized in Fig. 3). The best fit of a Gaussian distribution is shown in green.

Fig. S2. Pairwise interactions are sufficient to explain the patterns of microbial co-occurence. The parameters in our maximum entropy model were chosen to fit only the first and the second moments of the multivariate distribution of microbial abundances. Nevertheless, the model captures most of the higher-order correlations in the data suggesting pairwise interactions are sufficient to accurately describe the patterns of microbial co-occurences. **(A)** For each choice of three genera, the third order moment was computed by averaging the product of the log-abundances over all the samples in the IBD data ("observed") or from Eq. (17) ("predicted"), which states the predictions of the maximum entropy model. The plot shows excellent agreement between the two quantities. **(B)** For each choice of three genera ("index"), we plot the third-order central moment computed from the IBD data ("observed") and from an equally-sized sample drawn from our maximum entropy model ("Gaussian distribution"). The latter quantifies the expected deviations between the observations and predictions due to the finite size of the sample. **(C)** Same as (A), but for the fourth-order central moment. The expected level of noise is quantified via a sample from the maximum entropy model that obeys Eq. (17) exactly in the limit of infinite sample size. The correlation coefficient between "observed" and "predicted" values from this sample sets the upper bound on the expected correlation coefficient in IBD data.

Fig. S3. Microbial interactions are only weakly affected by host phenotype. To determine whether Crohn's disease drastically alters the pattern of microbial interactions, we computed and compared the covariance matrixes C^{CD} and C^{control} for CD and control groups respectively. The results of this calculation for IBD data are shown in blue. Each dot corresponds to a matrix element of C_{ij}, which is the covariance between the log-abundances of genera i and j. The x-coordinate is the covariance computed in the control group and the y-coordinate is the covariance computed in the CD group. To estimate the expected level of noise, we carried out the same analysis on two random partitions of the data that contain both controls and subjects with CD (shown in magenta). Since the groups are drawn from the same distribution, their covariance matrices must be identical on average. The spread of the magenta data points, therefore, sets the upper limit on the correlation coefficient between C^{CD} and C^{control}. We note, however, that this upper bound is unlikely to be reached for IBD data because some taxa have different noise levels in CD and control groups: eg. the taxa depleted in CD have a low abundance in this group and, therefore, higher error in the estimates of the correlation coefficients with other taxa. Overall, both IBD and partitioned data lie close to the diagonal and exhibit similar levels of variation. Thus, using the same covariance matrix for both CD and control groups is a reasonable first approximation. This approximation is valuable because it reduces the uncertainty in C_{ij} by allowing us to use the entire data to compute covariances and because it improves the stability of DAA to errors in C (see Fig. S12).

Fig. S4. Taxa directly associated with Crohn's disease. Note that the Green Genes database [116] used in QIIME [117] places Turicibacter under Erysipelotrichales and has a unique order of Turicibacterales. This apparent inconsistency may reflect insufficient understanding of Turicibacter phylogeny. The effect sizes and statistical significance are summarized in Tab. S3 and compared between DAA and conventional MWAS in Tab. S4.

Fig. S5. Comparison between correlations and direct interactions. The matrix of microbial interactions J is shown in **(A)** and the correlation matrix C is shown in **(B)**, which is the same as Fig. 1B of the main text. Both matrices are inferred from the IBD data set. Note that J is sparser than C. For greater clarity, the matrices are hierarchically clustered; therefore, the order of species in A and B is not the same.

Fig. S6. Comparison of networks inferred by Pearson correlation, SparCC, and DAA at the genus level. Three networks quantifying microbial co-occurrence or interactions have been inferred: one based on the Pearson correlation coefficient between log-abundances (which is closely related to the covariance matrix C), one using SparCC package from Ref. [56] that attempts to reduce compositional bias, and one based on the direct interactions J from DAA. In each network, we kept only links that were statistically different from 0 under a permutation test with 5% false discovery rate. The panels display Venn diagrams showing unique and overlapping links in these networks. All links are included in **(A)**, and the comparison is done irrespective of the sign of the link, i.e. agreement is reported even if one method reports a positive link and another method reports a negative link. In contrast, **(B)** and **(C)** show only positive and negative links respectively. Three conclusions can be drawn from these comparisons. First, the high overlap between SparCC and Pearson networks shows that log-transforms have largely accounted for the compositional bias. Second, all three methods agree on a large number of links suggesting that all methods are sensitive to some strong interactions. Third, DAA reports fewer links and identifies a few links not detected by other methods. This reflect the different nature of DAA links. While both Pearson correlation and SparCC infer correlation, which could be either direct or indirect (i.e. induced; see main text). DAA removes indirect correlations, thus reducing the total number of links, but also reveals pairwise interactions that could have been masked by strong correlations with a third species.

Fig. S7. The network based on the correlation coefficient between log-transformed abundances. We plotted the correlation-based network for the species detected by DAA. Note the similarities and differences with the interaction network shown in Fig. 3 of the main text. Only the links with the correlation coefficient greater than 0.27 or lower than -0.15 are shown, and all links are statistically significant ($q < 0.05$). All correlation coefficients and direct interactions are summarized in Tab. S6 for the genera and species detected by DAA.

Fig. S8. Direct associations retain full diagnostic power. The same as Fig. 4B of the main text, but for two other classifiers: random forest [65, 66] in **(A)** and support vector machine [67] in **(B)**.

Fig. S9. DAA detects all directly associated taxa in synthetic data, provided the sample size is sufficiently large. The same as Fig. 2A in the main text, but with the x-axis extended to larger sample sizes. Note that DAA recovers all 6 directly associated taxa when the sample size is greater than about 1200.

Fig. S10. Compositional bias has a negligible effect on DAA performance. All panels are the same as Fig. 2C in the main text, but with different normalization of the data prior to the analysis. **(A)** No normalization: the analysis is done on the counts from the OTU table, which do not add up to a constant number. **(B)** Total-sum scaling: The counts are converted into relative abundances by dividing by the total number of counts (reads) per sample. This plot is the same as Fig. 2C. **(C)** Centered-log ratio: First log-abundances were computed from unnormalized counts with a pseudocount of 1. Then, the mean log-abundances of the taxa was computed by averaging over the samples. Finally, the mean-log abundance of every taxon was subtracted from the log-abundances of this taxon in all samples. This procedure corresponds to normalizing by the geometric mean of the counts because it ensures that the mean log-abundance of a taxon is zero [55]. **(D)** Cumulative sum scaling: A normalization scheme proposed specifically for microbiome analyses was implemented following Ref. [59]. The results of the analyses in A-D are very similar suggesting that compositional bias does not lead to major artifacts. In particular, the number of associations in A grows at the same rate with the sample size as in B-D. This would not be the case if the compositional bias was strong because spurious associations due to normalization would lead to a greater number of detected taxa. Thus, we conclude that interspecific interactions rather than compositional effects are the primary source of spurious associations.

Fig. S11. The inference of the eigenvalues of the covariance matrix is robust to variation in sample size and bootstrapping. We repeatedly subsampled the IBD data set to half of its size and computed the eigenvalues of the covariance matrix C. The means and standard deviations from this bootstrap procedure are shown in green, and the eigenvalue inferred from the entire data are shown in black. The agreement between the different sample sizes and the small variation due to subsampling indicate that the spectral properties of C can be inferred quite accurately.

Fig. S12. Results of DAA are robust to variation in sample size and bootstrapping. Similar to Fig. S11, we repeatedly subsampled the IBD data set to half of its size and carried out DAA on each of the subsamples. **(A)** shows that there is a modest variation in inferred h. To a large extent, this variation is driven by the uncertainty in C and its inverse J. **(B)** shows a much smaller variation in Δh between control and CD groups (green symbols). The noise is reduced because, even though C changes from subsample to subsample, the same C is used to infer h for control and disease groups. Therefore, the variability in C has a much weaker effect on Δh. For comparison, we also show Δh obtained by bootstrapping the entire data set without preserving the diagnosis labels (black symbols). These data show the expected distribution of Δh under the null hypothesis of no associations. For genera detected by DAA, the black and the green error bars do not overlap suggesting that the results of DAA are not affected by the uncertainty in C and are robust to variation in sample size and bootstrapping.

Fig. S13. Results of DAA are not significantly affected by compositional effects. The quantity Δh between control and CD groups is the test statistic used to infer direct associations, and the variation of Δh due to sampling shows whether the statistical analysis is robust to small changes in the data set. To quantify these variations in Δh, we consider a sample drawn from the maximum entropy model fitted to the IBD data set and define two $\delta\Delta h$: one between normalized and not normalized sample and the other between the not normalized sample and the values of h in the maximum entropy model. The first $\delta\Delta h$ quantifies the variability due to normalization, while the second $\delta\Delta h$ quantifies the variability due to sampling. The plot shows the distribution of the absolute values of the difference between the absolute values of these $\delta\Delta h$ across genera for three normalization schemes: total-sum scaling (TSS), centered-log ratio (CLR) and cumulative sum scaling (CSS). The absolute Δh values of significant taxa in IBD RISK data (red rectangles) lie well outside of the distributions shown.

Fig. S14. Spurious associations in synthetic data with small and large effect sizes. The same analysis as in Fig. 2AB of the main text, but for synthetic data with smaller (A, B, C) and larger (D, E, F) effect sizes. **(A)** and **(D)** show the number of associations detected by traditional MWAS and DAA. **(B)** and **(E)** show the median effect sizes (median fold change) for the taxa detected by conventional MWAS. **(C)** and **(E)** show the effect sizes in both h and l for the taxa detected by DAA. The effect size for h was quantified as the relative percent difference in host-field between cases and controls, while the l-effect size was computed as described in the main text. Overall the results are similar to those in Fig. 2. In addition, (A) and (B) show that DAA can recover all directly associated taxa given a large number of samples without any false positives. For sample sizes exceeding 5000, DAA starts to detect indirect associations due to compositional effects.

Fig. S15. Sensitivity of DAA to eigenvalue threshold $\boldsymbol{\lambda_{\min}}$. Large $\lambda_{\min}$ retains only a few eigenvalues and imposes an artificially strong correlation structure on the data. As a result, DAA detects a large number of associations because it cannot distinguish direct from indirect effects. The performance of DAA improves as more eigenvalues are included and reaches a plateau. The dashed lines show the number of eigenvalues included for $\lambda_{\min} = 0.01$ used throughout our analysis. The insets show the eigenvalues of Λ in decreasing order.

Table S1. The list of genera used in the analysis. We included all genera that were present in more than 60% of either control or IBD subjects. The indices were chosen to hierarchically cluster the correlation matrix shown in Fig. 1b of the main text (index corresponds to the position of the genus on the x axis).

index	genus name	index	genus name	index	genus name
1	*[Prevotella]*	17	*Corynebacterium*	33	*Fusobacterium*
2	*Prevotella*	18	*Pseudomonas*	34	*Bacteroides*
3	*Dialister*	19	*Acinetobacter*	35	*Anaerostipes*
4	*Phascolarctobacterium*	20	*Erwinia*	36	*Parabacteroides*
5	*Epulopiscium*	21	*Actinomyces*	37	*[Eubacterium]*
6	*Eggerthella*	22	*Streptococcus*	38	*Odoribacter*
7	*Clostridium*	23	*Granulicatella*	39	*Oscillospira*
8	*Akkermansia*	24	*Neisseria*	40	*Lachnospira*
9	*Bilophila*	25	*Rothia*	41	*Roseburia*
10	*Bifidobacterium*	26	*Eikenella*	42	*Faecalibacterium*
11	*Collinsella*	27	*Campylobacter*	43	*Dorea*
12	*Sutterella*	28	*Veillonella*	44	*[Ruminococcus]*
13	*Parvimonas*	29	*Actinobacillus*	45	*Ruminococcus*
14	*Porphyromonas*	30	*Aggregatibacter*	46	*Blautia*
15	*Turicibacter*	31	*Haemophilus*	47	*Coprococcus*
16	*Staphylococcus*	32	*Holdemania*		

Table S2. Genera modified in synthetic data. Taxa indices are the same as in Table S1. Effect size is the percent change in the value of h.

**taxon index**	**effect size data 1 (main text)**	**effect size data 2 (small)**	**effect size data 3 (large)**
1	−18%	−17%	−44%
11	+24%	+14%	+129%
19	−36%	−12%	−72%
27	+17%	+16%	+67%
33	−13%	−14%	−28%
45	+18%	+13%	+112%

Table S3. Direct associations identified by DAA across phylogenetic levels.

**taxon name**	**direct effect, h_{CD}**	**direct effect, h_{ctrl}**	**difference, $\Delta h/\lvert h_{\mathrm{ctrl}}\rvert$**	**p-value**	**q-value**
		**Order level**			
Burkholderiales	−0.47	−0.66	+0.29	0.00013	0.0029
Turicibacterales	−1.7	−1.4	−0.18	0.00031	0.0036
Pasteurellales	−0.51	−0.69	+0.26	0.00068	0.0052
Campylobacterales	−1.6	−1.8	+0.1	0.00696	0.04
Erysipelotrichales	−2.5	−2.3	−0.083	0.0095	0.044
		**Family level**			
Alcaligenaceae	−0.68	−0.86	+0.21	0.00027	0.01
Clostridiaceae	−1.2	−0.99	−0.18	0.0026	0.049
Pasteurellaceae	−0.31	−0.47	+0.35	0.0033	0.049
		**Genus level**			
Roseburia	−1.2	−0.86	−0.35	0.000098	0.0046
Sutterella	−0.63	−0.80	+0.22	0.00043	0.01
Oscillospira	−2.4	−2.6	+0.097	0.0015	0.023
Turicibacter	+0.46	+0.69	−0.34	0.003	0.035
		**Species level**			
B.adolescentis	−0.23	+0.073	−4.12	0.00013	0.0037
E.dolichum	−0.51	−0.31	−0.65	0.0028	0.039
F.prausnitzii	−0.97	−0.81	−0.20	0.0042	0.039
A.segnis	−0.072	−0.25	+0.71	0.0056	0.04
B.producta	−0.75	−0.54	−0.38	0.0064	0.04

Table S4. Comparison between changes in h and in l for the taxa identified by DAA.

taxon name	abundance l_{CD}/l_{ctrl}	direct effect $\Delta h/\lvert h_{ctrl}\rvert$	q-value, l	q-value, h
		**Order level**		
Burkholderiales	+1.6	+0.29	0.04	0.0029
Turicibacterales	+0.45	−0.18	0.00002	0.0036
Pasteurellales	+4.2	+0.26	0	0.0052
Campylobacterales	+2.1	+0.1	0.000001	0.04
Erysipelotrichales	+0.34	−0.083	0	0.044
		**Family level**		
Alcaligenaceae	+1.7	+0.21	0.03	0.01
Clostridiaceae	+0.25	−0.18	0	0.049
Pasteurellaceae	+4.2	+0.35	0	0.049
		**Genus level**		
Roseburia	+0.21	−0.35	0	0.0046
Sutterella	+2.0	+0.22	0.004	0.01
Oscillospira	+0.84	+0.097	0.33	0.023
Turicibacter	+0.50	−0.34	0.0004	0.035
		**Species level**		
B.adolescentis	+0.43	−4.12	0.00004	0.0037
E.dolichum	+0.43	−0.65	0.00004	0.039
F.prausnitzii	+0.41	−0.20	0.000003	0.039
A.segnis	+2.8	+0.71	0	0.04
B.producta	+0.67	−0.38	0.03	0.04

Table S5. Indirect associations identified by uncorrected abundance analysis across phylogenetic levels.

taxon name	abundance, l_{CD}	abundance, l_{ctrl}	ratio, $l_{\mathrm{CD}}/l_{\mathrm{ctrl}}$	p-value	q-value
		**Order level**			
Erysipelotrichales	0.43	1.3	0.34	0	0
Clostridiales	18.4	31.1	0.59	0	0
Pasteurellales	1.2	0.29	4.2	0	0
Fusobacteriales	0.25	0.08	3.2	0	0
Enterobacteriales	2.8	0.81	3.4	0	0
Campylobacterales	0.017	0.008	2.1	0.000001	0.000004
Neisseriales	0.029	0.013	2.1	0.000002	0.000006
Turicibacterales	0.006	0.013	0.45	0.000008	0.00002
Bifidobacteriales	0.041	0.09	0.47	0.00004	0.0001
Bacteroidales	25.5	38.8	0.66	0.00008	0.00019
Gemellales	0.026	0.015	1.7	0.00023	0.00048
Verrucomicrobiales	0.017	0.036	0.48	0.0016	0.003
Sphingomonadales	0.010	0.007	1.4	0.02	0.04
Burkholderiales	1.3	0.86	1.6	0.02	0.04
		**Family level**			
Lachnospiraceae	4.9	11.5	0.42	0	0
Erysipelotrichaceae	0.44	1.3	0.34	0	0
Clostridiaceae	0.11	0.42	0.25	0	0
Pasteurellaceae	1.3	0.3	4.2	0	0
Fusobacteriaceae	0.25	0.08	3.3	0	0
Enterobacteriaceae	2.8	0.84	3.4	0	0.000001
Neisseriaceae	0.029	0.014	2.1	0.000002	0.00001
Ruminococcaceae	5.3	9.9	0.54	0.000002	0.00001
Turicibacteraceae	0.006	0.013	0.44	0.000006	0.00002
Bifidobacteriaceae	0.04	0.09	0.46	0.00003	0.0001
Campylobacteraceae	0.013	0.007	1.7	0.00012	0.0004
Christensenellaceae	0.007	0.01	0.55	0.00015	0.0005
Porphyromonadaceae	0.39	0.81	0.48	0.0002	0.0005
Gemellaceae	0.026	0.016	1.7	0.0003	0.0009
Bacteroidaceae	21.6	32.8	0.66	0.0004	0.001
Veillonellaceae	1.4	0.88	1.5	0.001	0.002
Verrucomicrobiaceae	0.018	0.038	0.47	0.001	0.003
Micrococcaceae	0.014	0.010	1.4	0.009	0.018
Alcaligenaceae	1.0	0.58	1.7	0.02	0.03
Prevotellaceae	0.04	0.07	0.58	0.02	0.04

**taxon name**	**abundance, l_{CD}**	**abundance, l_{ctrl}**	**ratio, l_{CD}/l_{ctrl}**	**p-value**	**q-value**
		**Genus level**			
Roseburia	0.042	0.20	0.21	0	0
Blautia	0.17	0.52	0.33	0	0
Aggregatibacter	0.11	0.022	5.0	0	0
Haemophilus	1.41	0.33	4.3	0	0
Lachnospira	0.022	0.076	0.29	0	0
Actinobacillus	0.025	0.009	2.7	0	0
Fusobacterium	0.36	0.10	3.7	0	0
Coprococcus	0.35	0.87	0.40	0	0
[Eubacterium]	0.048	0.13	0.36	0	0
Veillonella	0.30	0.13	2.2	0.000001	0.000006
Campylobacter	0.018	0.009	1.9	0.000002	0.000009
Eikenella	0.018	0.009	2.1	0.000002	0.000009
Neisseria	0.019	0.010	1.9	0.000002	0.000009
Faecalibacterium	1.92	4.27	0.45	0.000003	0.000009
Erwinia	0.016	0.009	1.9	0.000024	0.000076
Dialister	0.25	0.091	2.7	0.000035	0.0001
Holdemania	0.02	0.036	0.54	0.000039	0.0001
Turicibacter	0.008	0.017	0.5	0.00015	0.0004
[Ruminococcus]	0.57	0.91	0.62	0.00018	0.0004
Ruminococcus	0.57	0.91	0.62	0.00018	0.0004
Parabacteroides	0.44	0.91	0.49	0.0003	0.0008
Bifidobacterium	0.058	0.11	0.53	0.0007	0.001
Rothia	0.016	0.011	1.5	0.0008	0.002
Porphyromonas	0.018	0.010	1.7	0.001	0.002
Sutterella	1.46	0.73	2.0	0.002	0.004
Dorea	0.48	0.73	0.66	0.002	0.004
Bacteroides	1.22	41.9	0.75	0.005	0.01
Akkermansia	0.023	0.044	0.53	0.006	0.01
Anaerostipes	0.012	0.018	0.7	0.01	0.02
Staphylococcus	0.02	0.014	1.4	0.02	0.03
Granulicatella	0.034	0.024	1.4	0.02	0.03
Phascolarctobacterium	0.038	0.061	0.62	0.03	0.04
		**Species level**			
H. parainfluenzae	3.42	0.83	4.1	0	0
A. segnis	0.064	0.023	2.8	0	0
F. prausnitzii	5.0	12.3	0.41	0	0.000003
B. adolescentis	0.028	0.066	0.43	0.000005	0.00004
E. dolichum	0.10	0.23	0.44	0.000007	0.00004
V. parvula	0.06	0.033	1.82	0.00002	0.0001
V. dispar	0.51	0.27	1.91	0.0002	0.0008
N. subflava	0.041	0.025	1.62	0.0008	0.0027
Ros. faecis	0.023	0.035	0.65	0.0008	0.0027
P. copri	0.052	0.11	0.46	0.001	0.003
A. muciniphila	0.061	0.13	0.48	0.002	0.006
Bac. uniformis	0.71	1.2	0.58	0.012	0.027
R. mucilaginosa	0.039	0.028	1.39	0.015	0.031
Bl. producta	0.031	0.046	0.67	0.015	0.031
C. catus	0.045	0.067	0.67	0.021	0.039

Table S6. A summary of interaction strengths and log-abundance correlation coefficients for the core IBD network shown in Fig. 3 of the main text. Statistical significance was estimated by a permutation test. Specifically, we independently permuted the abundance of each taxa across samples and then computed the correlation and interaction matrices on the permuted data to generate the probability distribution for the null hypothesis of no interaction.

**interacting taxa**	**correlation strength, C_{ij}**	**interaction strength, J_{ij}**	**q-value, correlation**	**q-value, interaction**
A.segnis-B.producta	+0.16	+0.14	0.0011	0.0041
A.segnis-Oscillospira	−0.16	−0.17	0.0014	0.0011
A.segnis-Roseburia	−0.15	−0.19	0.0034	0.0006
A.segnis-Sutterella	−0.015	+0.046	0.80	0.41
A.segnis-Turicibacter	+0.18	+0.12	0	0.021
B.adolescentis-A.segnis	+0.19	+0.19	0	0.0006
B.adolescentis-B.producta	+0.26	+0.16	0	0.0019
B.adolescentis-Oscillospira	+0.069	−0.067	0.17	0.24
B.adolescentis-Roseburia	+0.25	+0.24	0	0
B.adolescentis-Sutterella	+0.036	+0.055	0.50	0.34
B.adolescentis-Turicibacter	+0.40	+0.46	0	0
B.producta-Oscillospira	+0.10	+0.04	0.044	0.47
B.producta-Roseburia	+0.100	+0.0063	0.047	0.92
B.producta-Sutterella	+0.0012	+0.092	0.98	0.091
B.producta-Turicibacter	+0.31	+0.23	0	0
E.dolichum-A.segnis	−0.0063	−0.027	0.92	0.66
E.dolichum-B.adolescentis	+0.19	+0.051	0.0002	0.35
E.dolichum-B.producta	+0.40	+0.46	0	0
E.dolichum-F.prausnitzii	+0.075	+0.0087	0.13	0.92
E.dolichum-Oscillospira	+0.27	+0.29	0	0
E.dolichum-Roseburia	+0.25	+0.21	0	0
E.dolichum-Sutterella	−0.080	−0.19	0.11	0
E.dolichum-Turicibacter	+0.20	+0.057	0	0.33
F.prausnitzii-A.segnis	−0.086	+0.0064	0.086	0.92
F.prausnitzii-B.adolescentis	+0.15	+0.20	0.0021	0
F.prausnitzii-B.producta	−0.065	−0.15	0.19	0.0032
F.prausnitzii-Oscillospira	+0.32	+0.29	0	0
F.prausnitzii-Roseburia	+0.35	+0.35	0	0
F.prausnitzii-Sutterella	+0.25	+0.204	0	0.0006
F.prausnitzii-Turicibacter	−0.095	−0.18	0.053	0.0003
Roseburia-Oscillospira	+0.29	+0.16	0	0.0034
Roseburia-Sutterella	+0.099	+0.019	0.05	0.76
Roseburia-Turicibacter	+0.099	+0.053	0.05	0.34
Sutterella-Oscillospira	+0.23	+0.24	0	0
Turicibacter-Oscillospira	+0.036	+0.076	0.50	0.18
Turicibacter-Sutterella	−0.12	−0.15	0.012	0.0026

References

[1] Turnbaugh PJ, Ley RE, Hamady M, Fraser-Liggett C, Knight R, Gordon JI. The human microbiome project: exploring the microbial part of ourselves in a changing world. Nature. 2007;449(7164):804.

[2] Yatsunenko T, Rey FE, Manary MJ, Trehan I, Dominguez-Bello MG, Contreras M, et al. Human gut microbiome viewed across age and geography. Nature. 2012;486(7402):222–227.

[3] Cho I, Blaser MJ. The human microbiome: at the interface of health and disease. Nature Reviews Genetics. 2012;13(4):260–270.

[4] Ding T, Schloss PD. Dynamics and associations of microbial community types across the human body. Nature. 2014;509(7500):357–360.

[5] Gilbert JA, Jansson JK, Knight R. The Earth Microbiome project: successes and aspirations. BMC biology. 2014;12(1):69.

[6] Bakken JS, Borody T, Brandt LJ, Brill JV, Demarco DC, Franzos MA, et al. Treating Clostridium difficile infection with fecal microbiota transplantation. Clinical Gastroenterology and Hepatology. 2011;9(12):1044–1049.

[7] Suez J, Korem T, Zeevi D, Zilberman-Schapira G, Thaiss CA, Maza O, et al. Artificial sweeteners induce glucose intolerance by altering the gut microbiota. Nature. 2014;514(7521):181–186.

[8] Jumpertz R, Le DS, Turnbaugh PJ, Trinidad C, Bogardus C, Gordon JI, et al. Energy-balance studies reveal associations between gut microbes, caloric load, and nutrient absorption in humans. The American journal of clinical nutrition. 2011;94(1):58–65.

[9] Turnbaugh PJ, Ley RE, Mahowald MA, Magrini V, Mardis ER, Gordon JI. An obesity-associated gut microbiome with increased capacity for energy harvest. nature. 2006;444(7122):1027–131.

[10] Messaoudi M, Lalonde R, Violle N, Javelot H, Desor D, Nejdi A, et al. Assessment of psychotropic-like properties of a probiotic formulation (Lactobacillus helveticus R0052 and Bifidobacterium longum R0175) in rats and human subjects. British Journal of Nutrition. 2011;105(05):755–764.

[11] Cryan JF, OMahony S. The microbiome-gut-brain axis: from bowel to behavior. Neurogastroenterology & Motility. 2011;23(3):187–192.

[12] Palm NW, De Zoete MR, Cullen TW, Barry NA, Stefanowski J, Hao L, et al. Immunoglobulin A coating identifies colitogenic bacteria in inflammatory bowel disease. Cell. 2014;158(5):1000–1010.

[13] Sampson TR, Debelius JW, Thron T, Janssen S, Shastri GG, Ilhan ZE, et al. Gut Microbiota Regulate Motor Deficits and Neuroinflammation in a Model of Parkinsons Disease. Cell. 2016;167(6):1469–1480.

[14] Qin J, Li Y, Cai Z, Li S, Zhu J, Zhang F, et al. A metagenome-wide association study of gut microbiota in type 2 diabetes. Nature. 2012;490(7418):55–60.

[15] Kostic AD, Gevers D, Siljander H, Vatanen T, Hyötyläinen T, Hämäläinen AM, et al. The dynamics of the human infant gut microbiome in development and in progression toward type 1 diabetes. Cell host & microbe. 2015;17(2):260–273.

[16] Giongo A, Gano KA, Crabb DB, Mukherjee N, Novelo LL, Casella G, et al. Toward defining the autoimmune microbiome for type 1 diabetes. The ISME journal. 2011;5(1):82–91.

[17] Brusca SB, Abramson SB, Scher JU. Microbiome and mucosal inflammation as extra-articular triggers for rheumatoid arthritis and autoimmunity. Current opinion in rheumatology. 2014;26(1):101.

[18] Taneja V. Arthritis susceptibility and the gut microbiome. FEBS letters. 2014;588(22):4244–4249.
[19] Williams BL, Hornig M, Parekh T, Lipkin WI. Application of novel PCR-based methods for detection, quantitation, and phylogenetic characterization of Sutterella species in intestinal biopsy samples from children with autism and gastrointestinal disturbances. MBio. 2012;3(1):e00261–11.
[20] Wang L, Christophersen CT, Sorich MJ, Gerber JP, Angley MT, Conlon MA. Increased abundance of Sutterella spp. and Ruminococcus torques in feces of children with autism spectrum disorder. Molecular autism. 2013;4(1):1.
[21] Gevers D, Kugathasan S, Denson LA, Vázquez-Baeza Y, Van Treuren W, Ren B, et al. The treatment-naive microbiome in new-onset Crohns disease. Cell Host 'I&' Microbe. 2014;15(3):382–392.
[22] El Mouzan M, Wang F, Al Mofarreh M, Menon R, Al Barrag A, Korolev KS, et al. Fungal Microbiota Profile in Newly Diagnosed Treatment-naïve Children with Crohns disease. Journal of Crohn's and Colitis. 2017; p. 1–7.
[23] Gilbert JA, Quinn RA, Debelius J, Xu ZZ, Morton J, Garg N, et al. Microbiome-wide association studies link dynamic microbial consortia to disease. Nature. 2016;535(7610):94–103.
[24] Son JS, Zheng LJ, Rowehl LM, Tian X, Zhang Y, Zhu W, et al. Comparison of fecal microbiota in children with autism spectrum disorders and neurotypical siblings in the Simons Simplex Collection. PloS ONE. 2015;10(10):e0137725.
[25] Wang F, Kaplan JL, Gold BD, Bhasin MK, Ward NL, Kellermayer R, et al. Detecting Microbial Dysbiosis Associated with Pediatric Crohn Disease Despite the High Variability of the Gut Microbiota. Cell Reports. 2016;14(4):945–955.
[26] De Cruz P, Prideaux L, Wagner J, Ng SC, McSweeney C, Kirkwood C, et al. Characterization of the gastrointestinal microbiota in health and inflammatory bowel disease. Inflammatory bowel diseases. 2012;18(2):372–390.
[27] Coyte KZ, Schluter J, Foster KR. The ecology of the microbiome: networks, competition, and stability. Science. 2015;350(6261):663–666.
[28] Rakoff-Nahoum S, Foster KR, Comstock LE. The evolution of cooperation within the gut microbiota. Nature. 2016;533(7602):255–259.
[29] Flint HJ, Duncan SH, Scott KP, Louis P. Interactions and competition within the microbial community of the human colon: links between diet and health. Environmental microbiology. 2007;9(5):1101–1111.
[30] Bashan A, Gibson TE, Friedman J, Carey VJ, Weiss ST, Hohmann EL, et al. Universality of human microbial dynamics. Nature. 2016;534(7606):259–262.
[31] Faust K, Sathirapongsasuti JF, Izard J, Segata N, Gevers D, Raes J, et al. Microbial co-occurrence relationships in the human microbiome. PLoS Comput Biol. 2012;8(7):e1002606.
[32] Magnúsdóttir S, Heinken A, Kutt L, Ravcheev DA, Bauer E, Noronha A, et al. Generation of genome-scale metabolic reconstructions for 773 members of the human gut microbiota. Nature Biotechnology. 2017;35:81–89.
[33] Chu J, Vila-Farres X, Inoyama D, Ternei M, Cohen LJ, Gordon EA, et al. Discovery of MRSA active antibiotics using primary sequence from the human microbiome. Nature Chemical Biology. 2016;12(12):1004–1006.
[34] Riley MA, Goldstone C, Wertz J, Gordon D. A phylogenetic approach to assessing the targets of microbial warfare. Journal of evolutionary biology. 2003;16(4):690–697.
[35] Czárán TL, Hoekstra RF, Pagie L. Chemical warfare between microbes promotes biodiversity. Proceedings of the National Academy of Sciences. 2002;99(2):786–790.

[36] Dethlefsen L, Eckburg PB, Bik EM, Relman DA. Assembly of the human intestinal microbiota. Trends in ecology & evolution. 2006;21(9):517–523.
[37] Mackie RI. Gut environment and evolution of mutualistic fermentative digestion. In: Gastrointestinal microbiology. Springer; 1997. p. 13–35.
[38] Stein RR, Marks DS, Sander C. Inferring pairwise interactions from biological data using maximum-entropy probability models. PLoS Comput Biol. 2015;11(7):e1004182.
[39] Bialek W. Biophysics: searching for principles. Princeton University Press; 2012.
[40] Morgan XC, Tickle TL, Sokol H, Gevers D, Devaney KL, Ward DV, et al. Dysfunction of the intestinal microbiome in inflammatory bowel disease and treatment. Genome biology. 2012;13(9):1.
[41] Machiels K, Joossens M, Sabino J, De Preter V, Arijs I, Eeckhaut V, et al. A decrease of the butyrate-producing species Roseburia hominis and Faecalibacterium prausnitzii defines dysbiosis in patients with ulcerative colitis. Gut. 2013; p. gutjnl–2013.
[42] Morgan XC, Tickle TL, Sokol H, Gevers D, Devaney KL, Ward DV, et al. Dysfunction of the intestinal microbiome in inflammatory bowel disease and treatment. Genome biology. 2012;13(9):1.
[43] Travis AJ, Kelly D, Flint HJ, Aminov RI. Complete genome sequence of the human gut symbiont Roseburia hominis. Genome announcements. 2015;3(6):e01286–15.
[44] Forbes JD, Van Domselaar G, Bernstein CN. The gut microbiota in immune-mediated inflammatory diseases. Frontiers in Microbiology. 2016;7:1081.
[45] Joossens M, Huys G, Cnockaert M, De Preter V, Verbeke K, Rutgeerts P, et al. Dysbiosis of the faecal microbiota in patients with Crohn's disease and their unaffected relatives. Gut. 2011;60(5):631–637.
[46] Sokol H, Seksik P, Furet J, Firmesse O, Nion-Larmurier I, Beaugerie L, et al. Low counts of Faecalibacterium prausnitzii in colitis microbiota. Inflammatory bowel diseases. 2009;15(8):1183–1189.
[47] Takahashi K, Nishida A, Fujimoto T, Fujii M, Shioya M, Imaeda H, et al. Reduced abundance of butyrate-producing bacteria species in the fecal microbial community in Crohn's disease. Digestion. 2016;93(1):59–65.
[48] Plischke M, Bergersen B. Equilibrium statistical physics. World Scientific Publishing Co Inc; 1994.
[49] Harte J. Maximum entropy and ecology: a theory of abundance, distribution, and energetics. Oxford University Press; 2011.
[50] Schneidman E, Berry MJ, Segev R, Bialek W. Weak pairwise correlations imply strongly correlated network states in a neural population. Nature. 2006;440(7087):1007–1012.
[51] Volkov I, Banavar JR, Hubbell SP, Maritan A. Inferring species interactions in tropical forests. Proceedings of the National Academy of Sciences. 2009;106(33):13854–13859.
[52] Mora T, Walczak AM, Del Castello L, Ginelli F, Melillo S, Parisi L, et al. Local equilibrium in bird flocks. Nature Physics. 2016;12(12):1153–1157.
[53] Morcos F, Pagnani A, Lunt B, Bertolino A, Marks DS, Sander C, et al. Direct-coupling analysis of residue coevolution captures native contacts across many protein families. Proceedings of the National Academy of Sciences. 2011;108(49):E1293–E1301.
[54] Dahirel V, Shekhar K, Pereyra F, Miura T, Artyomov M, Talsania S, et al. Coordinate linkage of HIV evolution reveals regions of immunological vulnerability. Proceedings of the National Academy of Sciences. 2011;108(28):11530–11535.
[55] Fisher CK, Mora T, Walczak AM. Variable habitat conditions drive species covariation in the human microbiota. PLOS Computational Biology. 2017;13(4):e1005435.
[56] Friedman J, Alm EJ. Inferring correlation networks from genomic survey data. PLoS com-

putational biology. 2012;8(9):e1002687.
[57] Aitchison J. The statistical analysis of compositional data. Chapman and Hall London; 1986.
[58] Pawlowsky-Glahn V, Buccianti A. Compositional data analysis: Theory and applications. John Wiley & Sons; 2011.
[59] Paulson JN, Stine O Colin, Bravo HC, Pop M. Differential abundance analysis for microbial marker-gene surveys. Nature methods. 2013;10(12):1200–1202.
[60] Egozcue JJ, Pawlowsky-Glahn V, Mateu-Figueras G, Barcelo-Vidal C. Isometric logratio transformations for compositional data analysis. Mathematical Geology. 2003;35(3):279–300.
[61] Benjamini Y, Hochberg Y. Controlling the false discovery rate: a practical and powerful approach to multiple testing. Journal of the royal statistical society Series B (Methodological). 1995; p. 289–300.
[62] Storey JD, Tibshirani R. Statistical significance for genomewide studies. Proceedings of the National Academy of Sciences. 2003;100(16):9440–9445.
[63] Power RA, Parkhill J, de Oliveira T. Microbial genome-wide association studies: lessons from human GWAS. Nature Reviews Genetics. 2016;.
[64] Voorman A, Lumley T, McKnight B, Rice K. Behavior of QQ-plots and genomic control in studies of gene-environment interaction. PloS one. 2011;6(5):e19416.
[65] Ho TK. Random decision forests. In: Document Analysis and Recognition, 1995., Proceedings of the Third International Conference on. vol. 1. IEEE; 1995. p. 278–282.
[66] Breiman L. Random forests. Machine learning. 2001;45(1):5–32.
[67] Cortes C, Vapnik V. Support-vector networks. Machine learning. 1995;20(3):273–297.
[68] Walker SH, Duncan DB. Estimation of the probability of an event as a function of several independent variables. Biometrika. 1967;54(1-2):167–179.
[69] Cox DR. The regression analysis of binary sequences. Journal of the Royal Statistical Society Series B (Methodological). 1958; p. 215–242.
[70] Tibshirani R. Regression shrinkage and selection via the lasso. Journal of the Royal Statistical Society Series B (Methodological). 1996; p. 267–288.
[71] Jostins L, Ripke S, Weersma RK, Duerr RH, McGovern DP, Hui KY, et al. Host-microbe interactions have shaped the genetic architecture of inflammatory bowel disease. Nature. 2012;491(7422):119–124.
[72] Xavier R, Podolsky D. Unravelling the pathogenesis of inflammatory bowel disease. Nature. 2007;448(7152):427–434.
[73] Sokol H, Pigneur B, Watterlot L, Lakhdari O, Bermúdez-Humarán LG, Gratadoux JJ, et al. Faecalibacterium prausnitzii is an anti-inflammatory commensal bacterium identified by gut microbiota analysis of Crohn disease patients. Proceedings of the National Academy of Sciences. 2008;105(43):16731–16736.
[74] Zhang M, Qiu X, Zhang H, Yang X, Hong N, Yang Y, et al. Faecalibacterium prausnitzii inhibits interleukin-17 to ameliorate colorectal colitis in rats. PloS one. 2014;9(10):e109146.
[75] Qiu X, Zhang M, Yang X, Hong N, Yu C. Faecalibacterium prausnitzii upregulates regulatory T cells and anti-inflammatory cytokines in treating TNBS-induced colitis. Journal of Crohn's and Colitis. 2013;7(11):e558–e568.
[76] Forbes JD, Van Domselaar G, Bernstein CN. The gut microbiota in immune-mediated inflammatory diseases. Frontiers in Microbiology. 2016;7.
[77] Scharek L, Hartmann L, Heinevetter L, Blaut M. Bifidobacterium adolescentis modulates the specific immune response to another human gut bacterium, Bacteroides thetaiotaomicron, in gnotobiotic rats. Immunobiology. 2000;202(5):429–441.
[78] Oyetayo VO, Oyetayo FL. Review-Potential of probiotics as biotherapeutic agents targeting the innate immune system. African Journal of Biotechnology. 2005;4(2):123–127.

[79] Duranti S, Milani C, Lugli GA, Mancabelli L, Turroni F, Ferrario C, et al. Evaluation of genetic diversity among strains of the human gut commensal Bifidobacterium adolescentis. Scientific reports. 2016;6.
[80] Sonomoto K, Yokota A. Lactic acid bacteria and bifidobacteria: current progress in advanced research. Horizon Scientific Press; 2011.
[81] Louis P, Flint HJ. Formation of propionate and butyrate by the human colonic microbiota. Environmental Microbiology. 2016;19:29–41.
[82] Jeraldo P, Hernandez A, Nielsen HB, Chen X, White BA, Goldenfeld N, et al. Capturing One of the Human Gut Microbiomes Most Wanted: Reconstructing the Genome of a Novel Butyrate-Producing, Clostridial Scavenger from Metagenomic Sequence Data. Frontiers in Microbiology. 2016;7.
[83] Carbonero F, Benefiel AC, Gaskins HR. Contributions of the microbial hydrogen economy to colonic homeostasis. Nature Reviews Gastroenterology and Hepatology. 2012;9(9):504–518.
[84] Louis P, Hold GL, Flint HJ. The gut microbiota, bacterial metabolites and colorectal cancer. Nature reviews Microbiology. 2014;12(10):661.
[85] Kettle H, Louis P, Holtrop G, Duncan SH, Flint HJ. Modelling the emergent dynamics and major metabolites of the human colonic microbiota. Environmental microbiology. 2015;17(5):1615–1630.
[86] Eeckhaut V, Van Immerseel F, Croubels S, De Baere S, Haesebrouck F, Ducatelle R, et al. Butyrate production in phylogenetically diverse Firmicutes isolated from the chicken caecum. Microbial biotechnology. 2011;4(4):503–512.
[87] Louis P, Flint HJ. Diversity, metabolism and microbial ecology of butyrate-producing bacteria from the human large intestine. FEMS microbiology letters. 2009;294(1):1–8.
[88] Gophna U, Konikoff T, Nielsen HB. Oscillospira and related bacteria–From metagenomic species to metabolic features. Environmental microbiology. 2017;19(3):835–841.
[89] Haberman Y, Tickle TL, Dexheimer PJ, Kim MO, Tang D, Karns R, et al. Pediatric Crohn disease patients exhibit specific ileal transcriptome and microbiome signature. The Journal of clinical investigation. 2014;124(8):3617.
[90] Kaakoush NO, Day AS, Huinao KD, Leach ST, Lemberg DA, Dowd SE, et al. Microbial dysbiosis in pediatric patients with Crohn's disease. Journal of clinical microbiology. 2012;50(10):3258–3266.
[91] Walters WA, Xu Z, Knight R. Meta-analyses of human gut microbes associated with obesity and IBD. FEBS letters. 2014;588(22):4223–4233.
[92] Verdam FJ, Fuentes S, de Jonge C, Zoetendal EG, Erbil R, Greve JW, et al. Human intestinal microbiota composition is associated with local and systemic inflammation in obesity. Obesity. 2013;21(12).
[93] Tims S, Derom C, Jonkers DM, Vlietinck R, Saris WH, Kleerebezem M, et al. Microbiota conservation and BMI signatures in adult monozygotic twins. The ISME journal. 2013;7(4):707.
[94] Zhu L, Baker SS, Gill C, Liu W, Alkhouri R, Baker RD, et al. Characterization of gut microbiomes in nonalcoholic steatohepatitis (NASH) patients: a connection between endogenous alcohol and NASH. Hepatology. 2013;57(2):601–609.
[95] Keren N, Konikoff FM, Paitan Y, Gabay G, Reshef L, Naftali T, et al. Interactions between the intestinal microbiota and bile acids in gallstones patients. Environmental microbiology reports. 2015;7(6):874–880.
[96] Milani C, Ticinesi A, Gerritsen J, Nouvenne A, Lugli GA, Mancabelli L, et al. Gut microbiota composition and Clostridium difficile infection in hospitalized elderly individuals: a metagenomic study. Scientific reports. 2016;6.
[97] Gu S, Chen Y, Zhang X, Lu H, Lv T, Shen P, et al. Identification of key taxa that favor

intestinal colonization of Clostridium difficile in an adult Chinese population. Microbes and infection. 2016;18(1):30–38.

[98] Minamoto Y, Otoni CC, Steelman SM, Büyükleblebici O, Steiner JM, Jergens AE, et al. Alteration of the fecal microbiota and serum metabolite profiles in dogs with idiopathic inflammatory bowel disease. Gut microbes. 2015;6(1):33–47.

[99] Werner T, Wagner SJ, Martínez I, Walter J, Chang JS, Clavel T, et al. Depletion of luminal iron alters the gut microbiota and prevents Crohn's disease-like ileitis. Gut. 2010; p. gut–2010.

[100] Presley LL, Wei B, Braun J, Borneman J. Bacteria associated with immunoregulatory cells in mice. Applied and environmental microbiology. 2010;76(3):936–941.

[101] Schwarz RS, Moran NA, Evans JD. Early gut colonizers shape parasite susceptibility and microbiota composition in honey bee workers. Proceedings of the National Academy of Sciences. 2016;113(33):9345–9350.

[102] Raja M, Fajar Ummer C. Aggregatibacter actinomycetemcomitans–A tooth killer? Journal of clinical and diagnostic research: JCDR. 2014;8(8):ZE13.

[103] Kamma J, Nakou M, Manti F. Predominant microflora of severe, moderate and minimal periodontal lesions in young adults with rapidly progressive periodontitis. Journal of periodontal research. 1995;30(1):66–72.

[104] Cassini M, Pilloni A, Condo S, Vitali L, Pasquantonio G, Cerroni L. Periodontal bacteria in the genital tract: are they related to adverse pregnancy outcome? International journal of immunopathology and pharmacology. 2013;26(4):931–939.

[105] Sokol H, Leducq V, Aschard H, Pham HP, Jegou S, Landman C, et al. Fungal microbiota dysbiosis in IBD. Gut. 2016; p. gutjnl–2015.

[106] Lavelle A, Lennon G, O'sullivan O, Docherty N, Balfe A, Maguire A, et al. Spatial variation of the colonic microbiota in patients with ulcerative colitis and control volunteers. Gut. 2015; p. gutjnl–2014.

[107] Mangin I, Bonnet R, Seksik P, Rigottier-Gois L, Sutren M, Bouhnik Y, et al. Molecular inventory of faecal microflora in patients with Crohn's disease. FEMS microbiology ecology. 2004;50(1):25–36.

[108] Gophna U, Sommerfeld K, Gophna S, Doolittle WF, van Zanten SJV. Differences between tissue-associated intestinal microfloras of patients with Crohn's disease and ulcerative colitis. Journal of clinical microbiology. 2006;44(11):4136–4141.

[109] Tyler AD, Knox N, Kabakchiev B, Milgrom R, Kirsch R, Cohen Z, et al. Characterization of the gut-associated microbiome in inflammatory pouch complications following ileal pouch-anal anastomosis. PloS one. 2013;8(9):e66934.

[110] Hansen R, Berry SH, Mukhopadhya I, Thomson JM, Saunders KA, Nicholl CE, et al. The microaerophilic microbiota of de-novo paediatric inflammatory bowel disease: the BISCUIT study. PLoS One. 2013;8(3):e58825.

[111] Hiippala K, Kainulainen V, Kalliomäki M, Arkkila P, Satokari R. Mucosal prevalence and interactions with the epithelium indicate commensalism of Sutterella spp. Frontiers in microbiology. 2016;7.

[112] Mukhopadhya I, Hansen R, Nicholl CE, Alhaidan YA, Thomson JM, Berry SH, et al. A comprehensive evaluation of colonic mucosal isolates of Sutterella wadsworthensis from inflammatory bowel disease. PLoS One. 2011;6(10):e27076.

[113] Biagi E, Candela M, Centanni M, Consolandi C, Rampelli S, Turroni S, et al. Gut microbiome in Down syndrome. PLoS one. 2014;9(11):e112023.

[114] Stewart GW. On the early history of the singular value decomposition. SIAM review. 1993;35(4):551–566.

[115] Pedregosa F, Varoquaux G, Gramfort A, Michel V, Thirion B, Grisel O, et al. Scikit-learn:

Machine learning in Python. Journal of Machine Learning Research. 2011;12(Oct):2825–2830.
[116] DeSantis TZ, Hugenholtz P, Larsen N, Rojas M, Brodie EL, Keller K et al. Greengenes, a chimera-checked 16S rRNA gene database and workbench compatible with ARB. Applied and environmental microbiology. 2006;72(7):5069-5072.
[117] Caporaso JG, Kuczynski J, Stombaugh J, Bittinger K, Bushman FD, Costello EK et al. QIIME allows analysis of high-throughput community sequencing data. Nature methods. 2010;1(May):335-336.
