# Supplementary figures and images for: Interactions between species introduce spurious associations in microbiome studies
Source: PLoS Comput Biol. 2018 Jan 16;14(1):e1005939. doi: 10.1371/journal.pcbi.1005939 (PMC5786326; doi:10.1371/journal.pcbi.1005939)

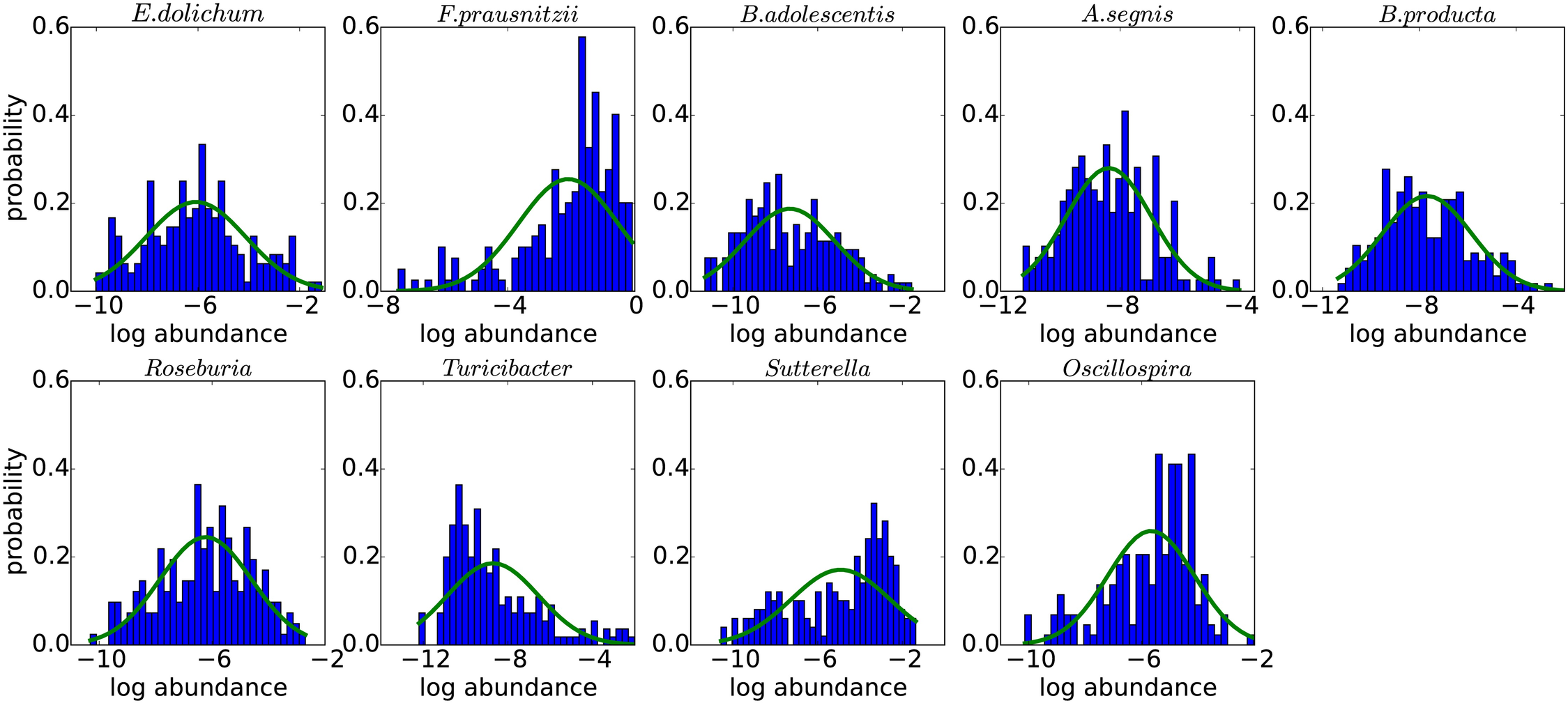

Supplement: S1 Fig — The histograms show probability distributions of the relative log-abundance for the species and genera detected by DAA (summarized in Fig 3). The best fit of a Gaussian distribution is shown in green. (TIF) [file pcbi.1005939.s002.tif]

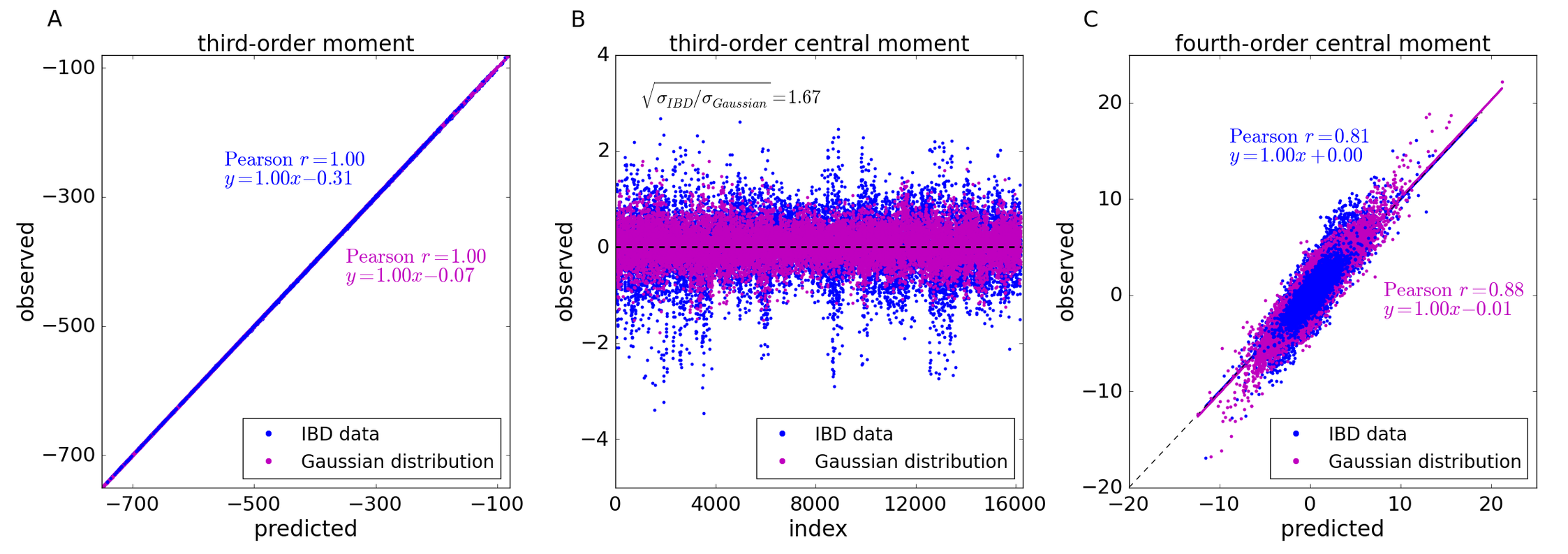

Supplement: S2 Fig — The parameters in our maximum entropy model were chosen to fit only the first and the second moments of the multivariate distribution of microbial abundances. Nevertheless, the model captures most of the higher-order correlations in the data suggesting pairwise interactions are sufficient to accurately describe the patterns of microbial co-occurences. (A) For each choice of three genera, the third order moment was computed by averaging the product of the log-abundances over all the samples in the IBD data (“observed”) or from Eq. (17) (“predicted”), which states the predictions of the maximum entropy model. The plot shows excellent agreement between the two quantities. (B) For each choice of three genera (“index”), we plot the third-order central moment computed from the IBD data (“observed”) and from an equally-sized sample drawn from our maximum entropy model (“Gaussian distribution”). The latter quantifies the expected deviations between the observations and predictions due to the finite size of the sample. (C) Same as (A), but for the fourth-order central moment. The expected level of noise is quantified via a sample from the maximum entropy model that obeys Eq. (17) exactly in the limit of infinite sample size. The correlation coefficient between “observed” and “predicted” values from this sample sets the upper bound on the expected correlation coefficient in IBD data. (TIF) [file pcbi.1005939.s003.tif]

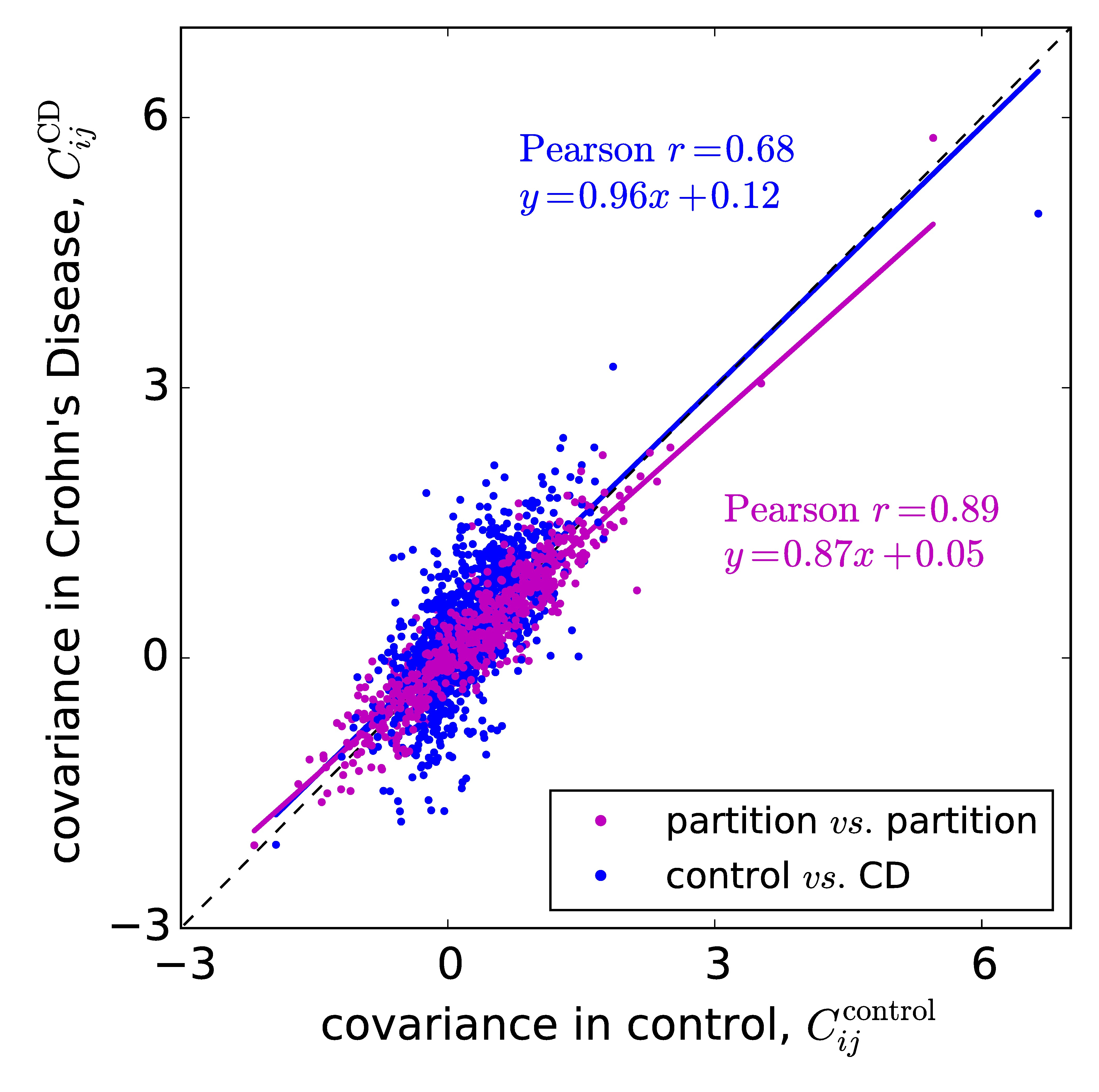

Supplement: S3 Fig — To determine whether Crohn’s disease drastically alters the pattern of microbial interactions, we computed and compared the covariance matrixes CCD and Ccontrol for CD and control groups respectively. The results of this calculation for IBD data are shown in blue. Each dot corresponds to a matrix element of Cij, which is the covariance between the log-abundances of genera i and j. The x-coordinate is the covariance computed in the control group and the y-coordinate is the covariance computed in the CD group. To estimate the expected level of noise, we carried out the same analysis on two random partitions of the data that contain both controls and subjects with CD (shown in magenta). Since the groups are drawn from the same distribution, their covariance matrices must be identical on average. The spread of the magenta data points, therefore, sets the upper limit on the correlation coefficient between CCD and Ccontrol. We note, however, that this upper bound is unlikely to be reached for IBD data because some taxa have different noise levels in CD and control groups: eg. the taxa depleted in CD have a low abundance in this group and, therefore, higher error in the estimates of the correlation coefficients with other taxa. Overall, both IBD and partitioned data lie close to the diagonal and exhibit similar levels of variation. Thus, using the same covariance matrix for both CD and control groups is a reasonable first approximation. This approximation is valuable because it reduces the uncertainty in Cij by allowing us to use the entire data to compute covariances and because it improves the stability of DAA to errors in C (see S12 Fig). (TIF) [file pcbi.1005939.s004.tif]

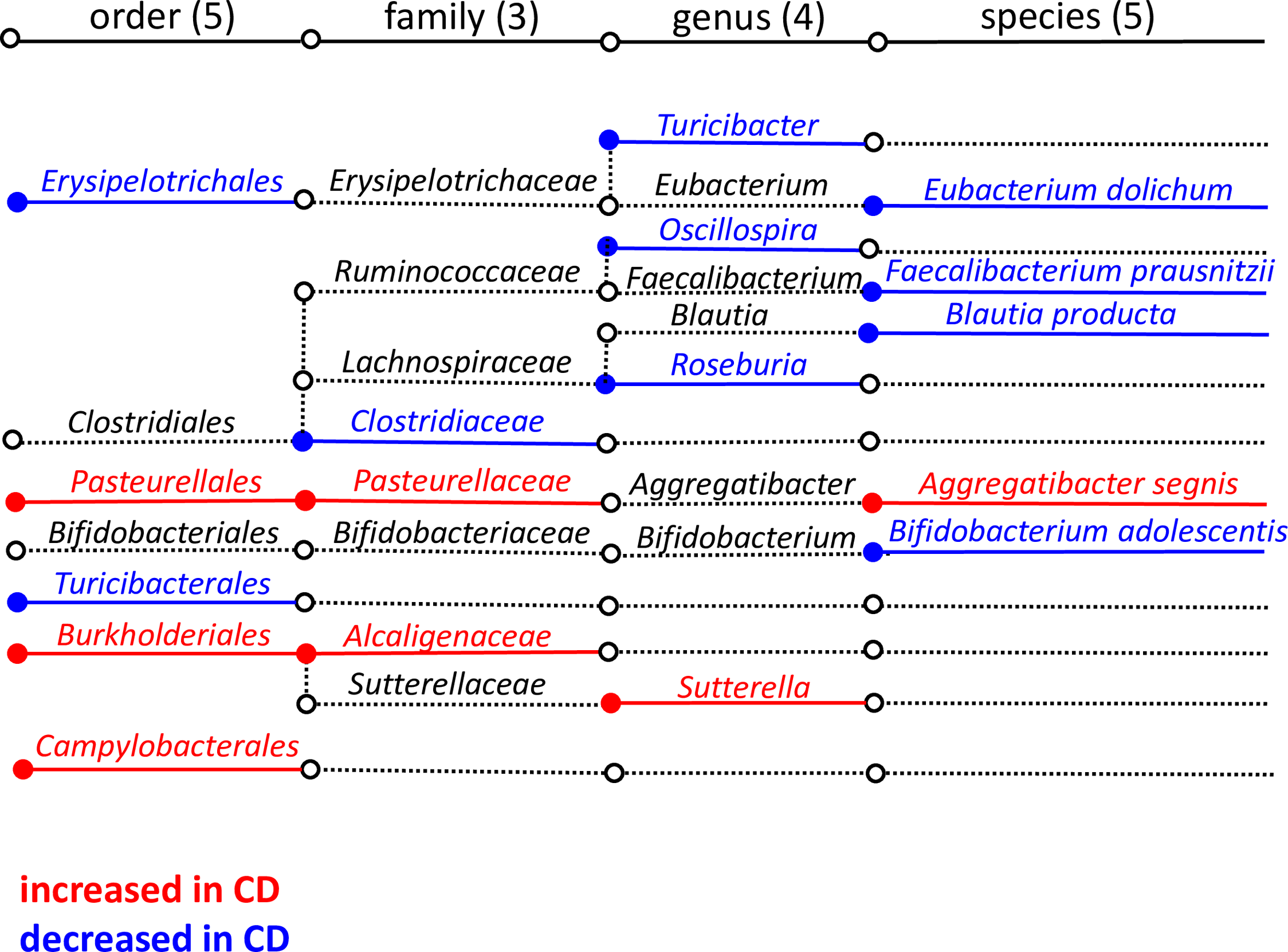

Supplement: S4 Fig — Note that the Green Genes database [112] used in QIIME [113] places Turicibacter under Erysipelotrichales and has a unique order of Turicibacterales. This apparent inconsistency may reflect insufficient understanding of Turicibacter phylogeny. The effect sizes and statistical significance are summarized and results for DAA and conventional MWAS are compared in S1 Text. (TIF) [file pcbi.1005939.s005.tif]

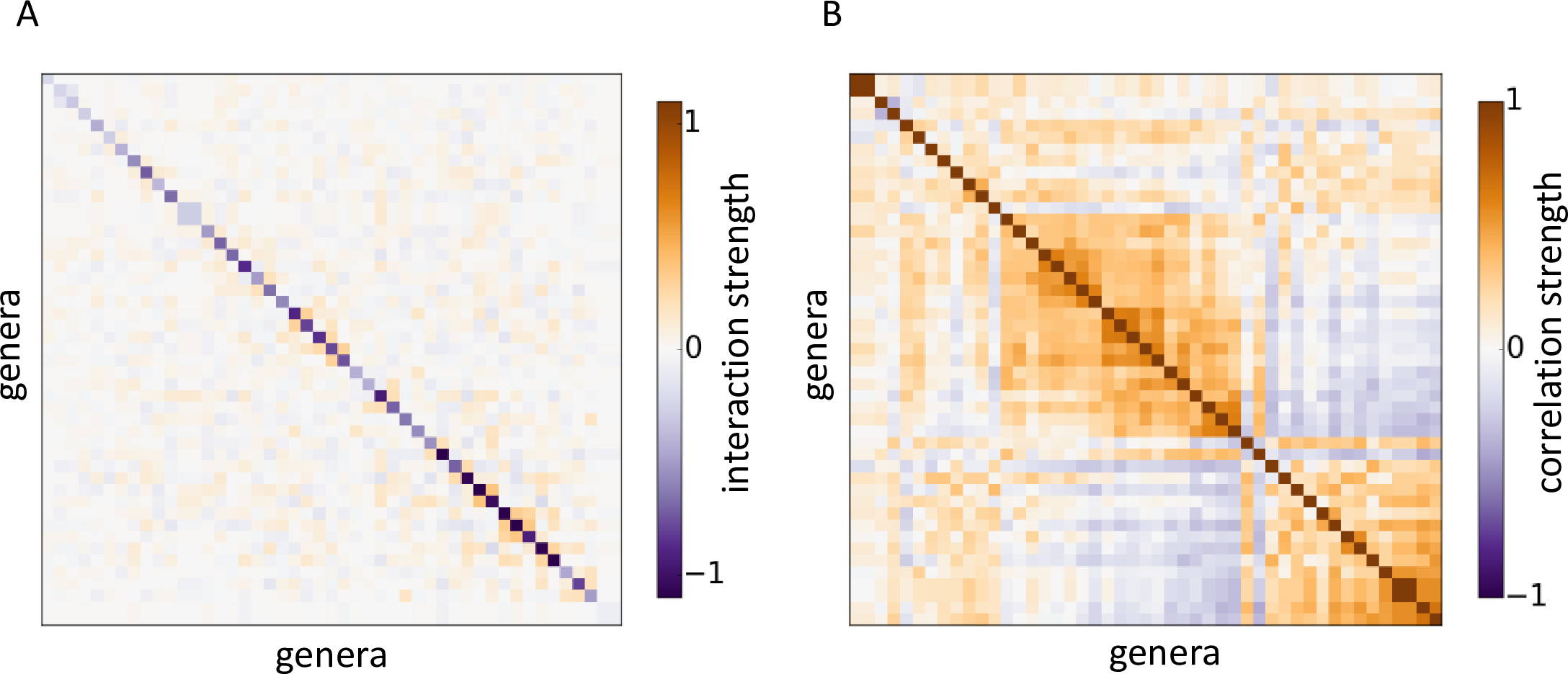

Supplement: S5 Fig — The matrix of microbial interactions J is shown in (A) and the correlation matrix C is shown in (B), which is the same as Fig 1B of the main text. Both matrices are inferred from the IBD data set. Note that J is sparser than C. For greater clarity, the matrices are hierarchically clustered; therefore, the order of species in (A) and (B) is not the same. (TIF) [file pcbi.1005939.s006.tif]

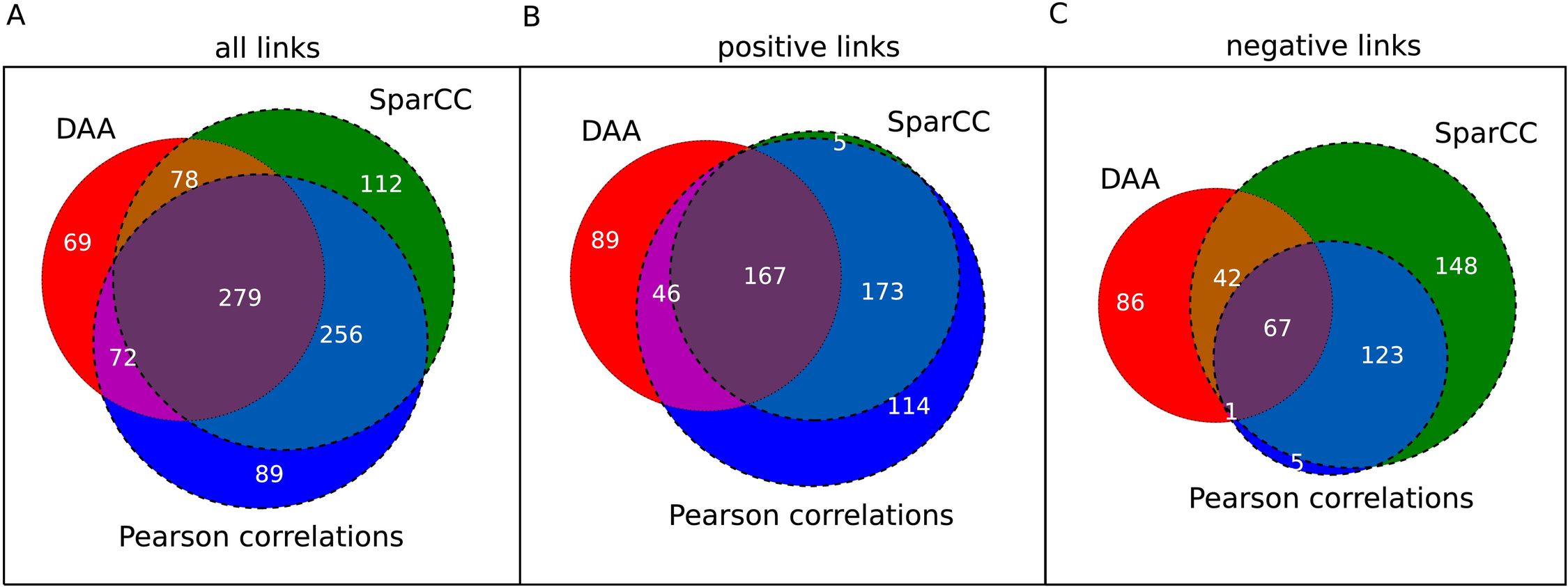

Supplement: S6 Fig — Three networks quantifying microbial co-occurrence or interactions have been inferred: one based on the Pearson correlation coefficient between log-abundances (which is closely related to the covariance matrix C), one using SparCC package from Ref. [56] that attempts to reduce compositional bias, and one based on the direct interactions J from DAA. In each network, we kept only links that were statistically different from 0 under a permutation test with 5% false discovery rate. The panels display Venn diagrams showing unique and overlapping links in these networks. All links are included in (A), and the comparison is done irrespective of the sign of the link, i.e. agreement is reported even if one method reports a positive link and another method reports a negative link. In contrast, (B) and (C) show only positive and negative links respectively. Three conclusions can be drawn from these comparisons. First, the high overlap between SparCC and Pearson networks shows that log-transforms have largely accounted for the compositional bias. Second, all three methods agree on a large number of links suggesting that all methods are sensitive to some strong interactions. Third, DAA reports fewer links and identifies a few links not detected by other methods. This reflect the different nature of DAA links. While both Pearson correlation and SparCC infer correlation, which could be either direct or indirect (i.e. induced; see main text). DAA removes indirect correlations, thus reducing the total number of links, but also reveals pairwise interactions that could have been masked by strong correlations with a third species. (TIF) [file pcbi.1005939.s007.tif]

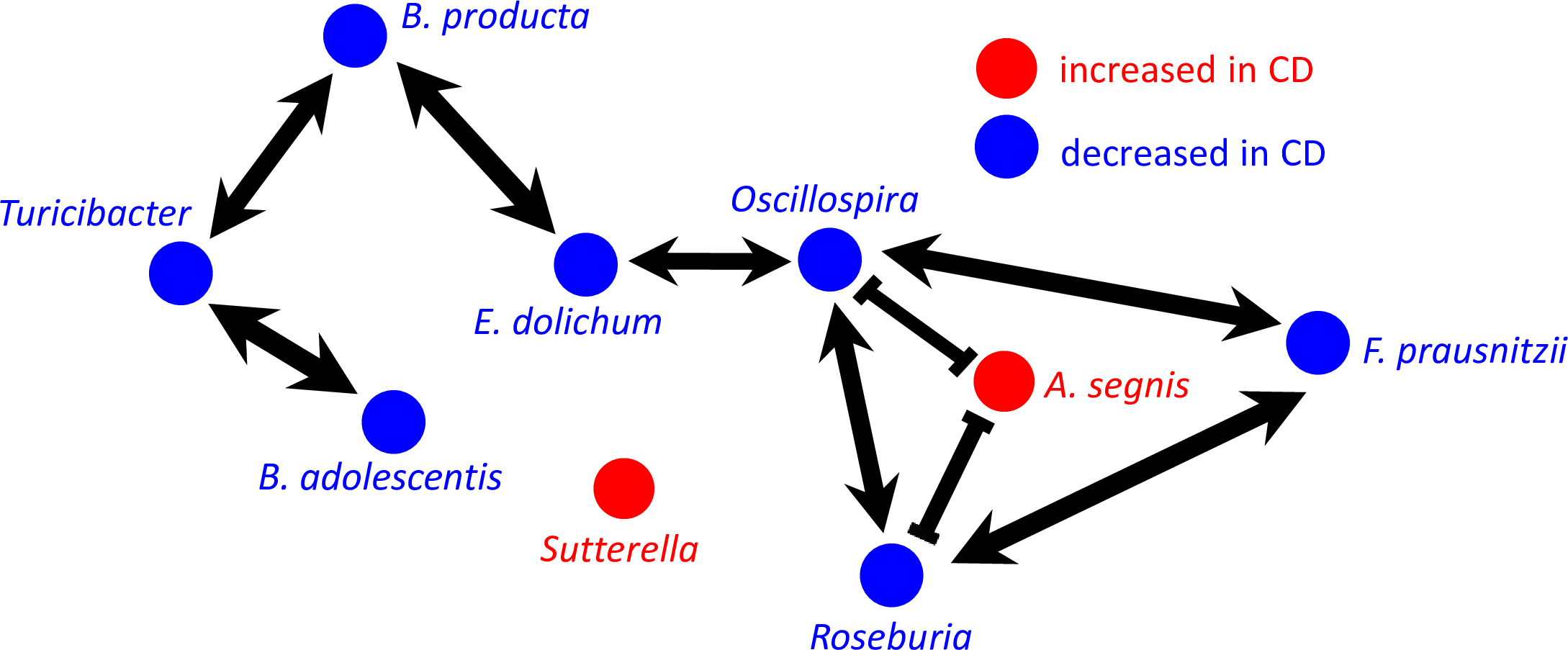

Supplement: S7 Fig — We plotted the correlation-based network for the species detected by DAA. Note the similarities and differences with the interaction network shown in Fig 3 of the main text. Only the links with the correlation coefficient greater than 0.27 or lower than -0.15 are shown, and all links are statistically significant (q < 0.05). All correlation coefficients and direct interactions are summarized in S1 Text for the genera and species detected by DAA. (TIF) [file pcbi.1005939.s008.tif]

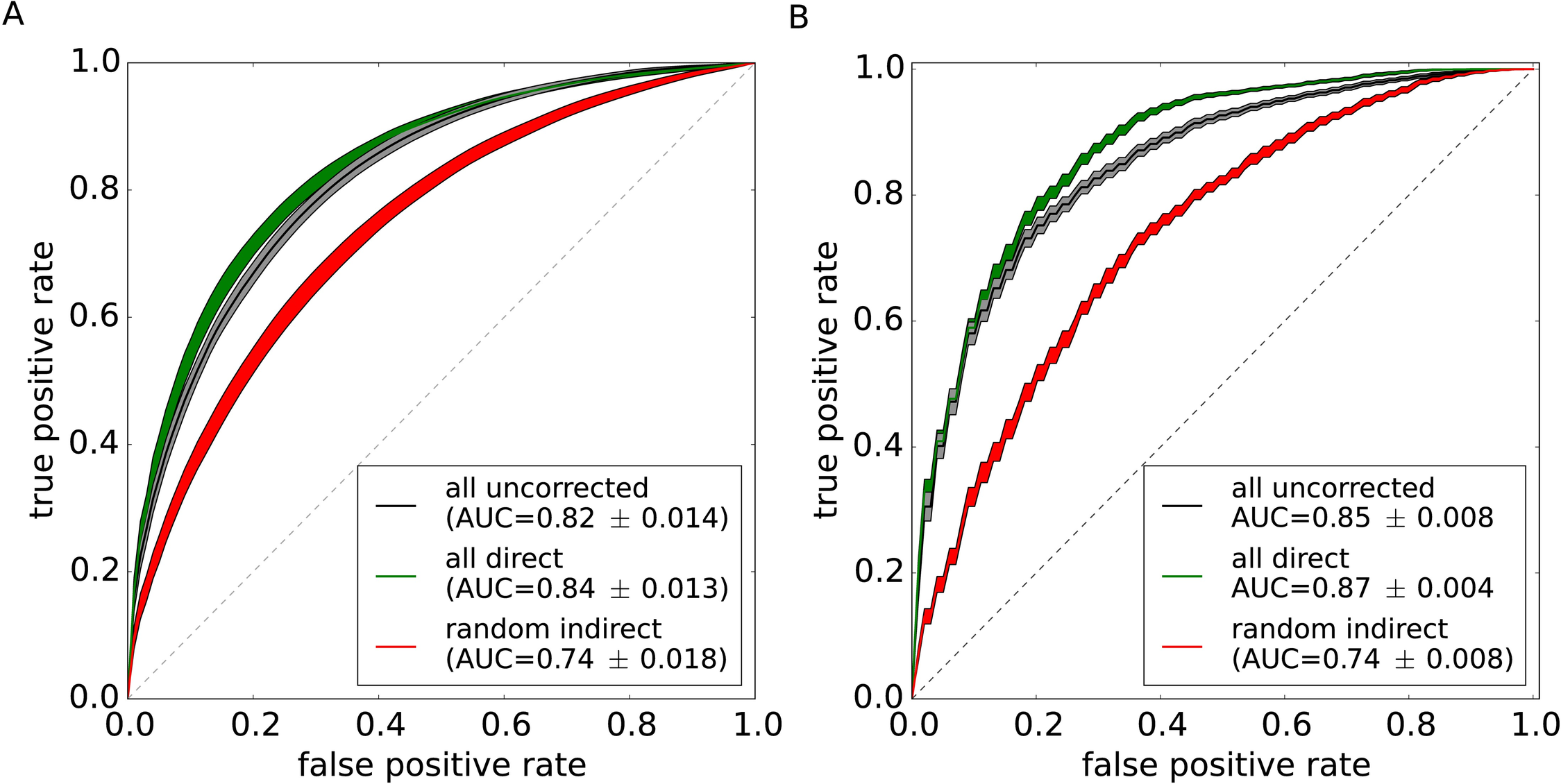

Supplement: S8 Fig — The same as Fig 4B of the main text, but for two other classifiers: random forest [64, 65] in (A) and support vector machine [66] in (B). (TIF) [file pcbi.1005939.s009.tif]

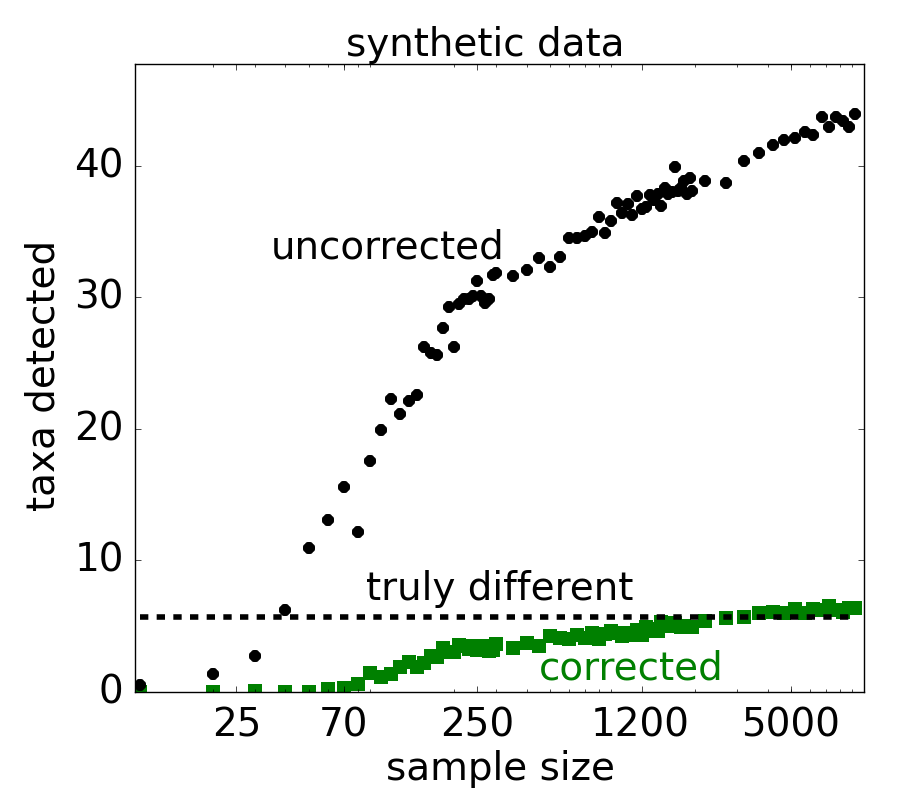

Supplement: S9 Fig — The same as Fig 2A in the main text, but with the x-axis extended to larger sample sizes. Note that DAA recovers all 6 directly associated taxa when the sample size is greater than about 1200. (TIF) [file pcbi.1005939.s010.tif]

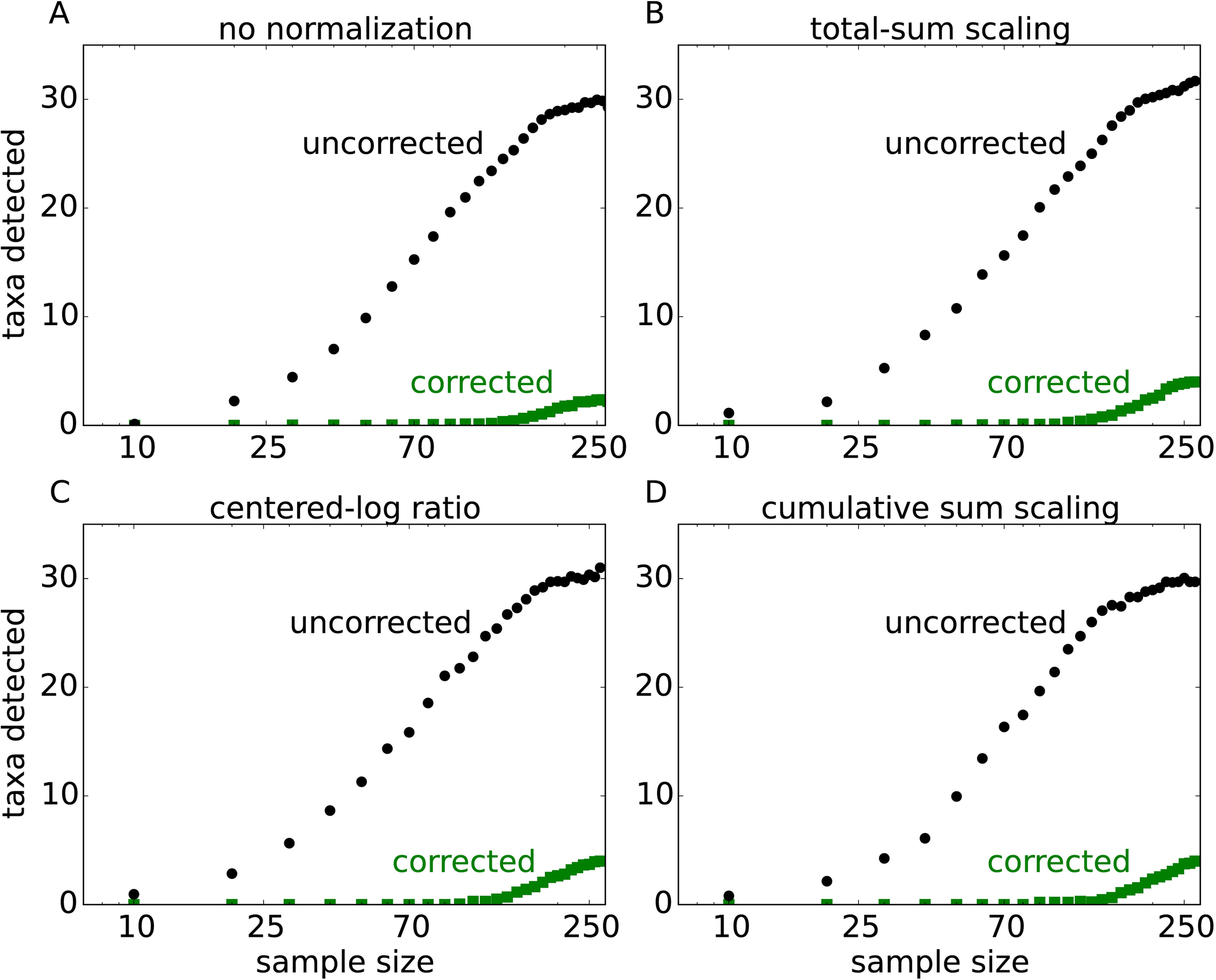

Supplement: S10 Fig — All panels are the same as Fig 2C in the main text, but with different normalization of the data prior to the analysis. (A) No normalization: the analysis is done on the counts from the OTU table, which do not add up to a constant number. (B) Total-sum scaling: The counts are converted into relative abundances by dividing by the total number of counts (reads) per sample. This plot is the same as Fig 2C. (C) Centered-log ratio: First log-abundances were computed from unnormalized counts with a pseudocount of 1. Then, the mean log-abundances of the taxa was computed by averaging over the samples. Finally, the mean-log abundance of every taxon was subtracted from the log-abundances of this taxon in all samples. This procedure corresponds to normalizing by the geometric mean of the counts because it ensures that the mean log-abundance of a taxon is zero [55]. (D) Cumulative sum scaling: A normalization scheme proposed specifically for microbiome analyses was implemented following Ref. [114]. The results of the analyses in (A)-(D) are very similar suggesting that compositional bias does not lead to major artifacts. In particular, the number of associations in (A) grows at the same rate with the sample size as in (B)-(D). This would not be the case if the compositional bias was strong because spurious associations due to normalization would lead to a greater number of detected taxa. Thus, we conclude that interspecific interactions rather than compositional effects are the primary source of spurious associations. (TIF) [file pcbi.1005939.s011.tif]

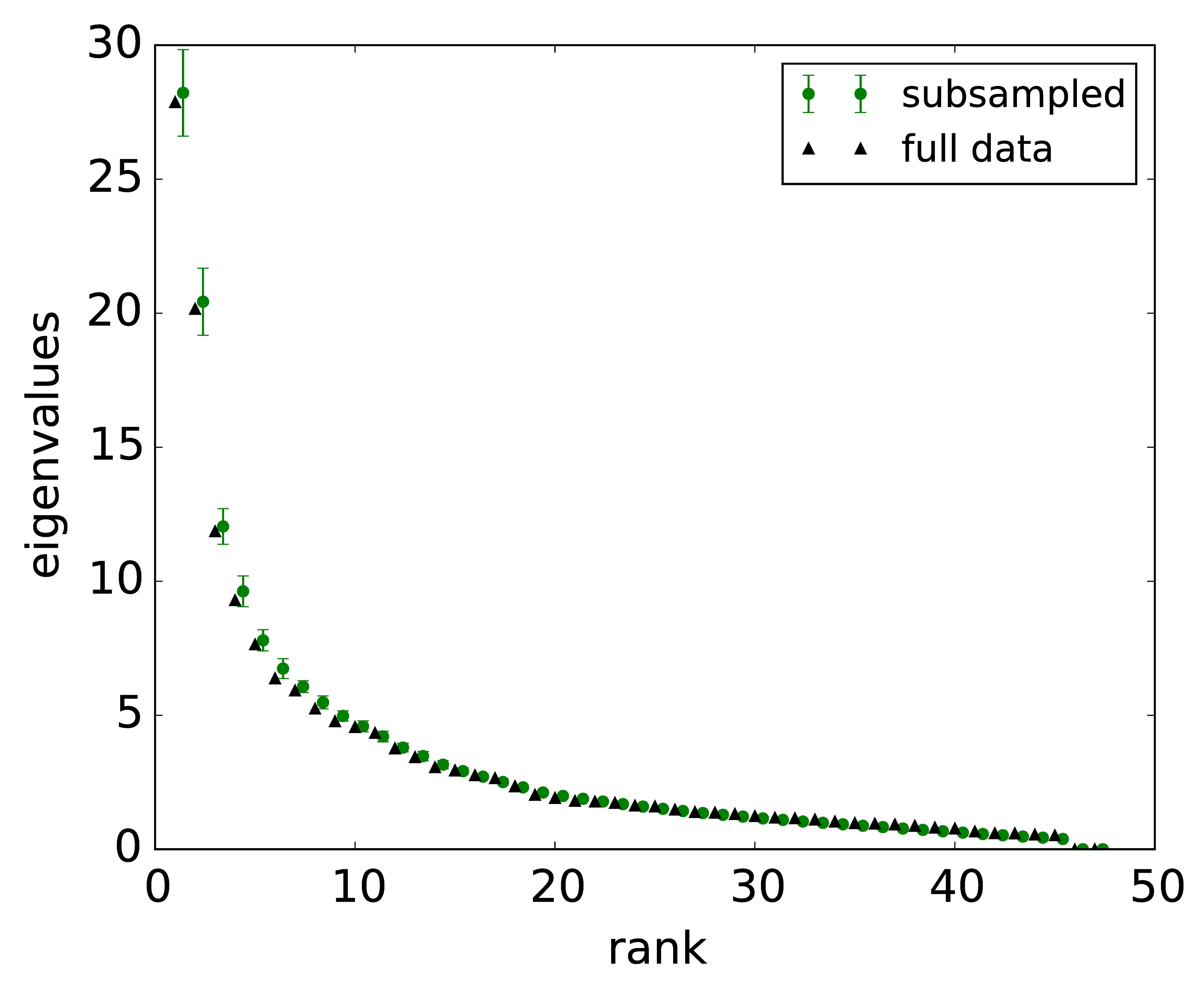

Supplement: S11 Fig — We repeatedly subsampled the IBD data set to half of its size and computed the eigenvalues of the covariance matrix C. The means and standard deviations from this bootstrap procedure are shown in green, and the eigenvalue inferred from the entire data are shown in black. The agreement between the different sample sizes and the small variation due to subsampling indicate that the spectral properties of C can be inferred quite accurately. (TIF) [file pcbi.1005939.s012.tif]

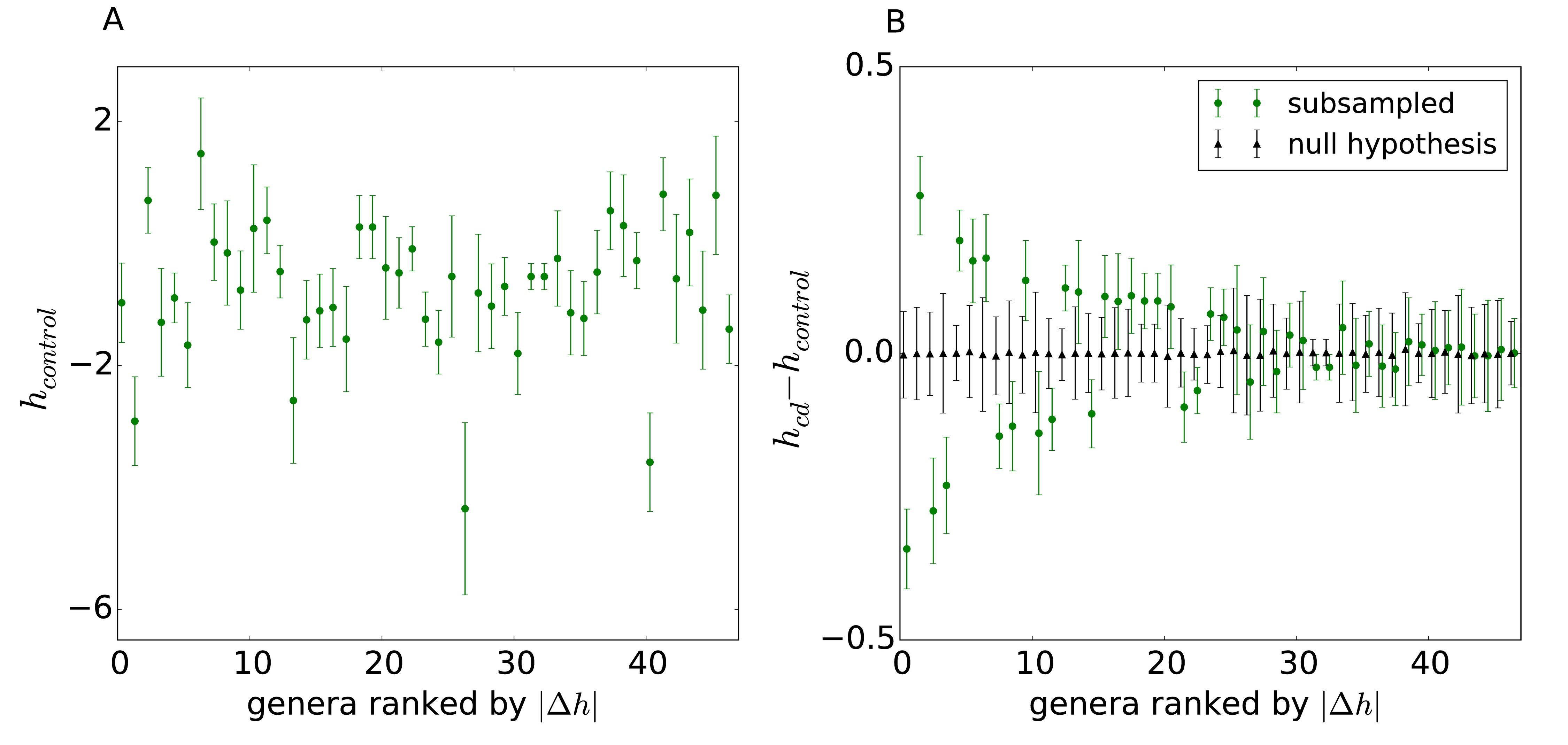

Supplement: S12 Fig — Similar to S11 Fig, we repeatedly subsampled the IBD data set to half of its size and carried out DAA on each of the subsamples. (A) shows that there is a modest variation in inferred h. To a large extent, this variation is driven by the uncertainty in C and its inverse J. (B) shows a much smaller variation in Δh between control and CD groups (green symbols). The noise is reduced because, even though C changes from subsample to subsample, the same C is used to infer h for control and disease groups. Therefore, the variability in C has a much weaker effect on Δh. For comparison, we also show Δh obtained by bootstrapping the entire data set without preserving the diagnosis labels (black symbols). These data show the expected distribution of Δh under the null hypothesis of no associations. For genera detected by DAA, the black and the green error bars do not overlap suggesting that the results of DAA are not affected by the uncertainty in C and are robust to variation in sample size and bootstrapping. (TIF) [file pcbi.1005939.s013.tif]

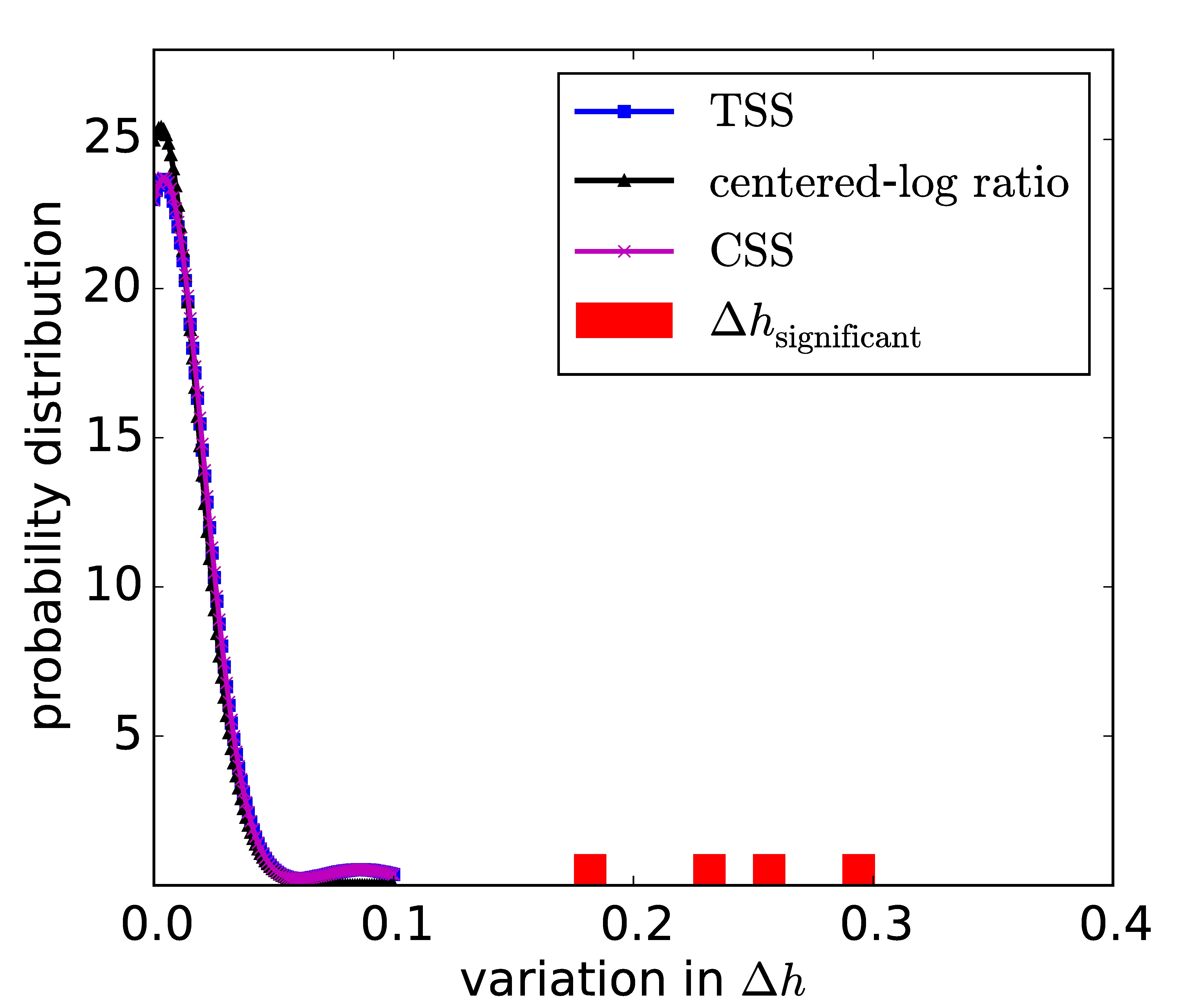

Supplement: S13 Fig — The quantity Δh between control and CD groups is the test statistic used to infer direct associations, and the variation of Δh due to sampling shows whether the statistical analysis is robust to small changes in the data set. To quantify these variations in Δh, we consider a sample drawn from the maximum entropy model fitted to the IBD data set and define two δΔh: one between normalized and not normalized sample and the other between the not normalized sample and the values of h in the maximum entropy model. The first δΔh quantifies the variability due to normalization, while the second δΔh quantifies the variability due to sampling. The plot shows the distribution of the absolute values of the difference between the absolute values of these δΔh across genera for three normalization schemes: total-sum scaling (TSS), centered-log ratio (CLR) and cumulative sum scaling (CSS). The absolute Δh values of significant taxa in IBD RISK data (red rectangles) lie well outside of the distributions shown. (TIF) [file pcbi.1005939.s014.tif]

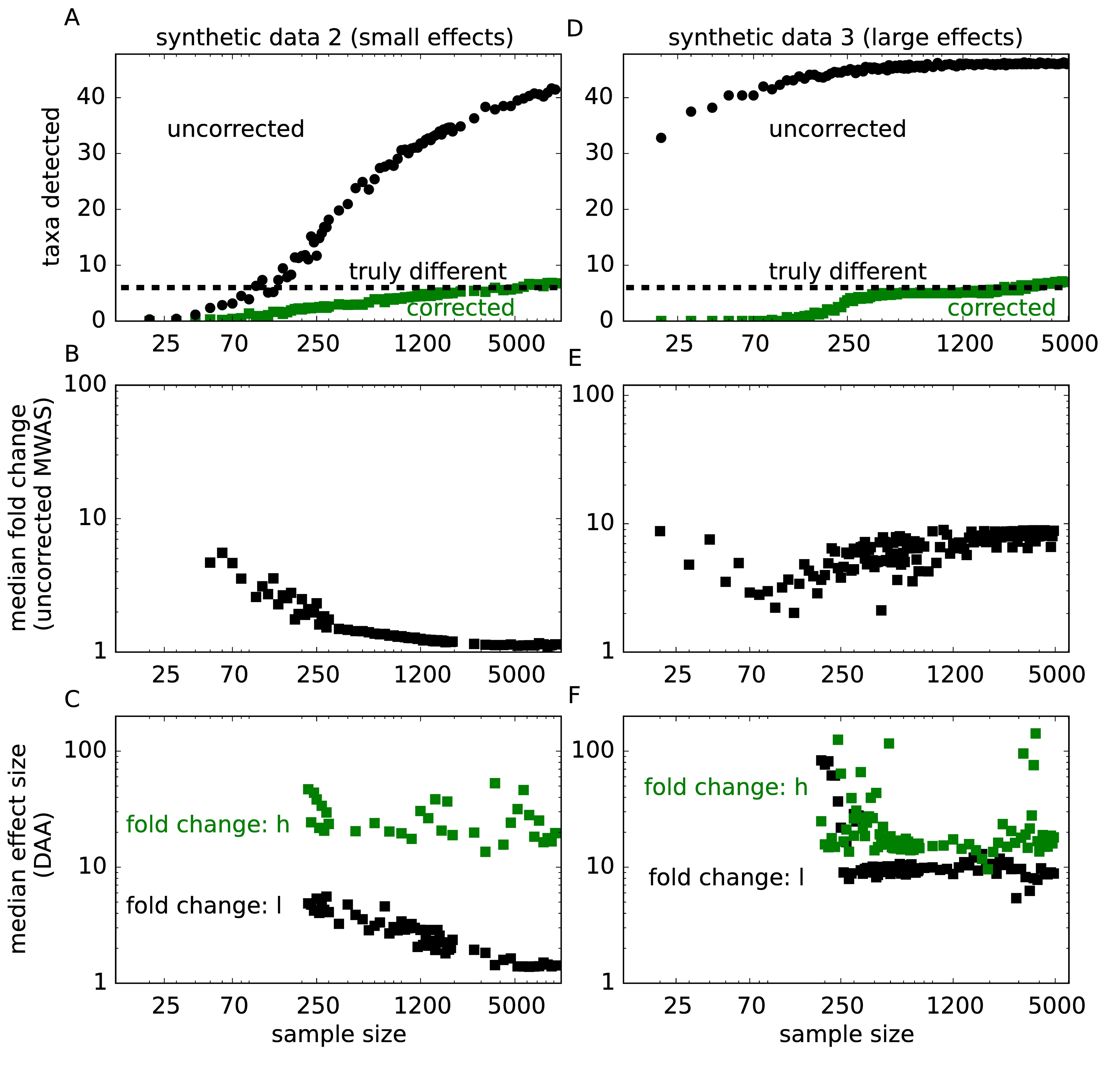

Supplement: S14 Fig — The same analysis as in Fig 2A and 2B of the main text, but for synthetic data with smaller (A, B, C) and larger (D, E, F) effect sizes. (A) and (D) show the number of associations detected by traditional MWAS and DAA. (B) and (E) show the median effect sizes (median fold change) for the taxa detected by conventional MWAS. (C) and (E) show the effect sizes in both h and l for the taxa detected by DAA. The effect size for h was quantified by the relative difference in h between cases and controls. The effect size for l was quantified as in (B) and (E). Overall the results are similar to those in Fig 2. In addition, (A) and (B) show that DAA can recover all directly associated taxa given a large number of samples without any false positives. For sample sizes exceeding 5000, DAA starts to detect indirect associations due to compositional effects. (TIF) [file pcbi.1005939.s015.tif]

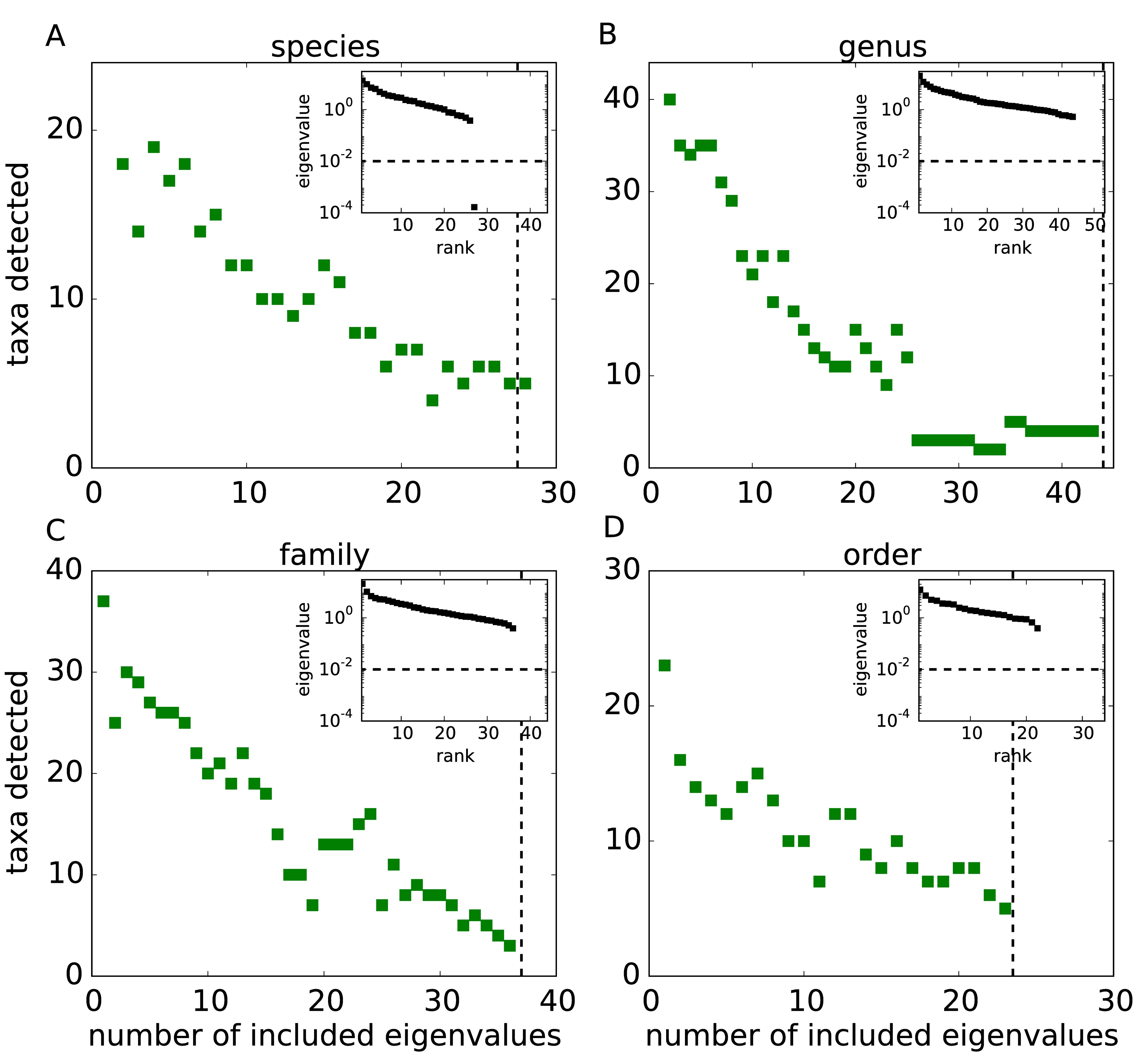

Supplement: S15 Fig — Large λmin retains only a few eigenvalues and imposes an artificially strong correlation structure on the data. As a result, DAA detects a large number of associations because it cannot distinguish direct from indirect effects. The performance of DAA improves as more eigenvalues are included and reaches a plateau. The dashed lines show the number of eigenvalues included for λmin = 0.01 used throughout our analysis. The insets show the eigenvalues of Λ in decreasing order. The four panels show the results for different taxonomic levels: from species to order. (TIF) [file pcbi.1005939.s016.tif]
